# Supplementary material for: Effects of body weight-supported Tai Chi Yunshou training on upper limb motor function in stroke patients: A three-arm parallel randomized controlled trial
Source: PLoS One. 2025 Jan 9;20(1):e0314025. doi: 10.1371/journal.pone.0314025 (PMC11717223; doi:10.1371/journal.pone.0314025)
Supplement: S1 Raw data — (DOCX) [file pone.0314025.s002.docx]

Raw data required to replicate the results

**Table 1** Comparison of baseline Chracteristics of the three groups of patients

| Chracteristics | CRT+BWS-TCY (n=29) | CRT (n=31) | CRT+RAT  (n=30) |
| --- | --- | --- | --- |
|  |  |  |  |
| Age, mean±SD | 59.41±11.78 | 63.13±12.51 | 65.44±13.36 |
| Gender (male, %) | 23 (67.6%) | 25 (73.5%) | 20 (58.8%) |
| Duration of disease (days) | 62.83±29.85 | 63.23±32.58 | 65.00±24.46 |
| Type of disease (Ischemic, %) | 21 (72.4%) | 21 (67.7%) | 22 (73.3%) |
| Hemiplegic side (Lelf, %) | 8 (27.6%) | 13 (41.9%) | 5 (16.7%) |
| Handedness (Right, %) | 27 (93.1%) | 29 (93.5%) | 28 (93.3%) |
| Heart rate (beats/minute) | 80.10±8.17 | 75.19±6.87 | 79.17±9.51 |
| Systolic blood pressure (mmHg) | 139.45±12.4 | 136.77±16.8 | 134.73±16.6 |
| Diastolic blood pressure (mmHg) | 84.45±7.49 | 79.39±9.59 | 81.60±9.41 |
| NIHSS, mean±SD | 15.66±3.55 | 16.26±3.10 | 15.17±3.14 |
| Brunnstrom staging | | | |
| Phase Ⅰ  Phase Ⅱ  Phase Ⅲ  Phase Ⅳ | 5 (17.2%)  10 (34.5%)  11 (37.9%)  3 (10.3%) | 3(9.7%)  11 (35.5%)  15 (48.4%)  2 (6.5%) | 2 (6.7%)  10 (33.3%)  12 (40.0%)  6 (20.0%) |
| Upper limb manual muscle strength test | | | |
| Level 0 | 0 (0%) | 1 (3.2%) | 0 (0%) |
| Level 1 | 4 (13.8%) | 6 (19.4%) | 3 (10.0%) |
| Level 2 | 13 (44.8%) | 18 (58.1%) | 21 (70.0%) |
| Level 3 | 12 (41.4%) | 6 (19.4%) | 6 (20.0%) |
| FMA-UE (0–66, score) | 25.28±9.27 | 25.09±9.56 | 27.73±10.76 |
| WMFT (0–75, score) | 29.17±8.68 | 29.13±6.99 | 27.97±7.52 |
| AEE (0-90, °) | 14.87±4.59 | 15.61±4.76 | 15.88±5.02 |
| MBI (0–100, score) | 46.38±14.01 | 47.74±9.99 | 48.33±10.61 |
| SS-QOL(49–245, score) | 99.79±13.81 | 99.58±12.39 | 99.37±13.02 |
| Upper limb joint movement angle | | | |
| Shoulder flexion | 45±20.92 | 51.50±25.18 | 49.35±22.89 |
| Shoulder extension | 17.93±9.28 | 19.58±8.99 | 19.33±9.66 |
| Shoulder abduction | 37.14±30.75 | 33.42±25.93 | 40.37±34.44 |
| Shoulder adduction | 14.21±7.98 | 13.16±7.01 | 15.23±9.74 |
| Shoulder external rotation | 20.97±12.18 | 18.00±11.51 | 22.40±13.38 |
| Shoulder internal rotation | 24.52±13.15 | 21.32±12.29 | 26.13±14.51 |
| Elbow flexion | 28.97±29.49 | 27.26±24.73 | 33.00±32.85 |
| Forearm pronation | 28.59±4.09 | 25.68±12.96 | 30.03±14.35 |
| Forearm supination | 20.62±12.85 | 18.42±11.46 | 21.93±13.38 |

| **Table 2** Repeated measurement of Mauchly spherical test for upper limb motor function | | | | | |
| --- | --- | --- | --- | --- | --- |
| Outcome measures | Mauchly W value | Approximate chi-square value | Significance | Degrees of freedom | Greenhouse-Greisser correction |
| FMA-UE | 0.160 | 157.208 | <0.001 | 5 | 0.496 |
| WMFT | 0.236 | 123.722 | <0.001 | 5 | 0.537 |

| **Table 3** Repeated measures analysis of variance for upper limb motor function | | | | | | | |
| --- | --- | --- | --- | --- | --- | --- | --- |
| Outcome measures | Scourses | Sum of Squares | Degrees of freedom | Mean Square | *F* value | Significance | Eta Squared |
| FMA-UE | Time | 19020.42 | 1.487 | 7499.84 | 285.87 | <0.001 | 0.77 |
|  | Group×Time | 1279.92 | 2.957 | 357.57 | 9.62 | <0.001 | 0.18 |
|  | Group | 1955.29 | 2 | 1313.66 | 3.67 | 0.030 | 0.08 |
| WMFT | Time | 18916.43 | 1.612 | 11735.78 | 392.52 | <0.001 | 0.82 |
|  | Group×Time | 839.88 | 3.224 | 260.53 | 8.71 | <0.001 | 0.17 |
|  | Group | 1878.65 | 2 | 939.33 | 4.25 | 0.017 | 0.09 |

**Table 4** Ratings of upper limb motor function in three groups at different time points

| Outcome measures | Groups | Time point | Mean | Standard deviation | Confidence Interval (%) | |
| --- | --- | --- | --- | --- | --- | --- |
|  |  |  |  |  | Upper | Lower |
| FMA-UE | CRT+BWS-TCY | before intervention | 25.28 | 9.27 | 21.75 | 28.80 |
|  |  | 4 weeks after intervention | 35.62 | 8.70 | 32.31 | 38.93 |
|  |  | 8 weeks after intervention | 42.48 | 8.70 | 39.17 | 45.79 |
|  |  | 12 weeks after intervention | 52.45 | 8.91 | 49.06 | 55.84 |
|  | CRT | before intervention | 25.10 | 9.56 | 32.31 | 38.93 |
|  |  | 4 weeks after intervention | 32.16 | 8.69 | 28.97 | 35.35 |
|  |  | 8 weeks after intervention | 35.26 | 8.82 | 32.02 | 38.49 |
|  |  | 12 weeks after intervention | 40.65 | 10.27 | 36.88 | 44.41 |
|  | CRT+RAT | before intervention | 27.73 | 10.76 | 23.71 | 31.75 |
|  |  | 4 weeks after intervention | 34.83 | 8.34 | 31.72 | 37.95 |
|  |  | 8 weeks after intervention | 39.00 | 8.43 | 35.85 | 42.15 |
|  |  | 12 weeks after intervention | 48.93 | 6.51 | 46.50 | 51.36 |
| WMFT | CRT+BWS-TCY | before intervention | 29.17 | 8.68 | 25.93 | 32.41 |
|  |  | 4 weeks after intervention | 34.73 | 8.56 | 28.59 | 33.61 |
|  |  | 8 weeks after intervention | 43.20 | 9.49 | 32.36 | 37.64 |
|  |  | 12 weeks after intervention | 49.10 | 8.58 | 45.89 | 52.31 |
|  | CRT | before intervention | 29.13 | 6.99 | 25.93 | 32.41 |
|  |  | 4 weeks after intervention | 31.10 | 6.73 | 28.59 | 33.61 |
|  |  | 8 weeks after intervention | 35.00 | 7.07 | 32.36 | 37.64 |
|  |  | 12 weeks after intervention | 38.43 | 7.93 | 35.47 | 41.39 |
|  | CRT+RAT | before intervention | 27.97 | 7.52 | 25.16 | 30.78 |
|  |  | 4 weeks after intervention | 33.17 | 7.19 | 30.48 | 35.85 |
|  |  | 8 weeks after intervention | 43.00 | 6.06 | 40.74 | 45.26 |
|  |  | 12 weeks after intervention | 48.97 | 5.57 | 46.89 | 51.05 |

**Table 5** Differences in upper limb motor function between the three groups after the intervention and before the intervention (Mean±SD, score)

| Outcome measures | Groups | Difference between 4 weeks after intervention and before intervention | Difference between 8 weeks after intervention and before intervention | Difference between 12 weeks after intervention and before intervention |
| --- | --- | --- | --- | --- |
| FMA-UE | CRT+BWS-TCY | 10.34±8.84 | 17.21±9.17 | 27.17±9.64 |
|  | CRT | 7.06±8.33 | 10.16±8.71 | 15.55±9.04 |
|  | CRT+RAT | 8.13±8.3 | 11.27±7.29 | 17.30±9.26 |
| WMFT | CRT+BWS-TCY | 8.59±7.41 | 15.69±7.87 | 24.45±6.89 |
|  | CRT | 6.29±7.99 | 9.32±8.42 | 14.81±8.73 |
|  | CRT+RAT | 7.03±7.00 | 12.37±5.28 | 20.30±6.57 |

**Table 6** Comparison of upper limb motor function scores among the three groups after 12 weeks of intervention

| Outcome measures | Comparison between groups | t value | *P* value (two-tailed) | Mean Difference | Standard error value |
| --- | --- | --- | --- | --- | --- |
| FMA-UE | CRT+BWS-TCY *VS* CRT | 4.740 | <0.001 | 11.80 | 2.49 |
|  | CRT+BWS-TCY *VS* CRT+RAT | 1.802 | 0.077 | 4.39 | 2.44 |
|  | CRT+RAT *VS* CRT | 3.242 | 0.002 | 7.41 | 2.89 |
| WMFT | CRT+BWS-TCY VS CRT | 4.720 | <0.001 | 9.64 | 2.02 |
|  | CRT+BWS-TCY VS CRT+RAT | 2.365 | 0.021 | 4.15 | 1.75 |
|  | CRT+RAT VS CRT | 2.770 | 0.007 | 5.49 | 1.97 |

**Table 7** Repeated measurement of Mauchly sphericity test for AEE

| Mauchly W value | Approximate chi-square value | Significance | Degrees of freedom | Greenhouse-Greisser correction |
| --- | --- | --- | --- | --- |
| 0.454 | 67.699 | <0.001 | 5 | 0.683 |

**Table 8** Repeated measures analysis of variance for AEE

| Scourses | Sum of Squares | Degrees of freedom | Mean Square | F value | Significance | Eta Squared |
| --- | --- | --- | --- | --- | --- | --- |
| Time | 1531.696 | 1.564 | 979.036 | 351.425 | <0.001 | 0.802 |
| Group×Time | 108.682 | 3.129 | 34.734 | 12.521 | <0.001 | 0.224 |
| Group | 510.554 | 2 | 255.277 | 4.372 | 0.016 | 0.091 |

**Table 9** Angle conditions of three groups of AEE at different time points

| Groups | Time point | Mean | Standard deviation | Confidence interval (%) | |
| --- | --- | --- | --- | --- | --- |
|  |  |  |  | Upper | Lower |
| CRT+BWS-TCY | before intervention | 15.138 | 4.26 | 13.52 | 16.76 |
|  | 4 weeks after intervention | 11.41 | 3.84 | 9.95 | 12.87 |
|  | 8 weeks after intervention | 8.714 | 3.48 | 7.39 | 10.04 |
|  | 12 weeks after intervention | 7.776 | 2.99 | 6.64 | 8.91 |
| CRT | before intervention | 15.755 | 4.61 | 14.06 | 17.45 |
|  | 4 weeks after intervention | 14.023 | 4.72 | 12.29 | 15.76 |
|  | 8 weeks after intervention | 12.771 | 4.02 | 11.29 | 14.24 |
|  | 12 weeks after intervention | 11.726 | 3.87 | 10.31 | 13.14 |
| CRT+RAT | before intervention | 15.98 | 4.62 | 14.26 | 17.70 |
|  | 4 weeks after intervention | 13.523 | 3.88 | 12.07 | 14.97 |
|  | 8 weeks after intervention | 11.80 | 3.77 | 10.39 | 13.21 |
|  | 12 weeks after intervention | 10.283 | 3.09 | 9.13 | 11.44 |

**Table 10** Differences between AEE in three groups after intervention and before intervention (Mean±SD, °)

| Groups | Difference between 4 weeks after intervention and before intervention | Difference between 8 weeks after intervention and before intervention | Difference between 12 weeks after intervention and before intervention |
| --- | --- | --- | --- |
| CRT+BWS-TCY | -3.73±1.77 | -6.42±3.01 | -7.36±3.05 |
| CRT | -1.73±0.87 | -2.98±1.36 | -4.03±1.65 |
| CRT+RAT | -2.46±1.21 | -4.18±1.55 | -5.69±2.16 |

**Table 11** Comparison of AEE among three groups after 12 weeks of intervention

| Comparison between groups | t value | *P* value (two-tailed) | Mean Difference | Standard error value |
| --- | --- | --- | --- | --- |
| CRT+BWS-TCY *VS* CRT | -4.404 | <0.001 | -3.95 | 0.89 |
| CRT+BWS-TCY *VS* CRT+RAT | -3.164 | 0.002 | -2.51 | 0.79 |
| CRT+RAT *VS* CRT | -1.606 | 0.114 | -1.442 | 0.89 |

**Table 12** Repeated measurement of Mauchly spherical test for the maximum range of motion of the upper limbs

| Outcome measures | Mauchly W value | Approximate chi-square value | Significance | Degrees of freedom | Greenhouse-Greisser correction |
| --- | --- | --- | --- | --- | --- |
| Shoulder flexion | 0.198 | 138.845 | <0.001 | 5 | 0.526 |
| Shoulder extension | 0.057 | 246.235 | <0.001 | 5 | 0.411 |
| Shoulder abduction | 0.157 | 158.530 | <0.001 | 5 | 0.492 |
| Shoulder adduction | 0.170 | 151.833 | <0.001 | 5 | 0.504 |
| Shoulder external rotation | 0.125 | 177.979 | <0.001 | 5 | 0.479 |
| Shoulder internal rotation | 0.143 | 166.911 | <0.001 | 5 | 0.512 |
| Elbow flexion | 0.097 | 199.743 | <0.001 | 5 | 0.461 |
| Forearm pronation | 0.253 | 76.582 | <0.001 | 5 | 0.531 |
| Forearm supination | 0.106 | 192.163 | <0.001 | 5 | 0.454 |

**Table 13** Repeated measures analysis of variance for upper limb joint mobility

| Outcome measures | Scourses | Sum of Squares | Degrees of freedom | Mean Square |
| --- | --- | --- | --- | --- |
| Shoulder flexion | Time | 131997.529 | 303.560 | ＜0.001 |
|  | Group | 32683.145 | 4.883 | 0.010 |
|  | Group×Time | 13745.799 | 15.806 | ＜0.001 |
| Shoulder extension | Time | 8655.856 | 177.055 | ＜0.001 |
|  | Group | 152.737 | 0.229 | 0.796 |
|  | Group×Time | 236.172 | 2.415 | 0.082 |
| Shoulder abduction | Time | 149861.850 | 345.13 | ＜0.001 |
|  | Group | 28084.535 | 4.256 | 0.017 |
|  | Group×Time | 1163.772 | 12.855 | ＜0.001 |
| Shoulder adduction | Time | 11011.475 | 360.313 | ＜0.001 |
|  | Group | 974.023 | 2.927 | 0.059 |
|  | Group×Time | 478.975 | 1.683 | 0.192 |
| Shoulder external rotation | Time | 40501.941 | 470.021 | ＜0.001 |
|  | Group | 2017.778 | 2.182 | 0.119 |
|  | Group×Time | 453.458 | 2.631 | 0.055 |
| Shoulder internal rotation | Time | 38839.871 | 363.660 | ＜0.001 |
|  | Group | 1720.211 | 3.537 | 0.033 |
|  | Group×Time | 1344.688 | 6.295 | ＜0.001 |
| Elbow flexion | Time | 170538.522 | 404.103 | ＜0.001 |
|  | Group | 16362.363 | 2.783 | 0.067 |
|  | Group×Time | 6933.046 | 8.214 | ＜0.001 |
| Forearm pronation | Time | 37400.553 | 268.468 | ＜0.001 |
|  | Group | 2364.103 | 2.263 | 0.110 |
|  | Group×Time | 787.238 | 2.825 | 0.045 |
| Forearm supination | Time | 45228.957 | 383.234 | ＜0.001 |
|  | Group | 1296.778 | 1.401 | 0.252 |
|  | Group×Time | 635.845 | 2.694 | 0.055 |

**Table 14** Maximum range of motion of upper limb joints in three groups at different time points (Mean ± SD, °)

| Outcome measures | Groups | before intervention | weeks after intervention | weeks after intervention | 12 weeks after intervention |
| --- | --- | --- | --- | --- | --- |
| Shoulder flexion | CRT+BWS-TCY | 37.45±30.52 | 63.86±28.69^*^ | 81.14±28.65^*^ | 112.97±34.73^*#^ |
|  | CRT | 33.77±25.93 | 47.19±25.36 | 55.45±26.09 | 68.48±31.83 |
|  | CRT+RAT | 44.63±32.54 | 59.17±32.12 | 72.83±34.19^*^ | 92.00±36.32^*^ |
| Shoulder extension | CRT+BWS-TCY | 17.93±9.28 | 25.21±9.73 | 29.86±10.73 | 33.93±14.08 |
|  | CRT | 19.58±8.99 | 23.03±8.48 | 27.06±7.84 | 30.87±7.70 |
|  | CRT+RAT | 19.33±9.66 | 24.30±9.17 | 28.27±9.53 | 31.57±10.86 |
| Shoulder abduction | CRT+BWS-TCY | 37.14±30.75 | 65.69±28.56^*^ | 82.69±28.59^*^ | 113.31±33.23^*#^ |
|  | CRT | 33.42±25.93 | 50.10±26.03 | 58.55±26.69 | 72.39±32.74 |
|  | CRT+RAT | 40.37±34.44 | 62.20±30.64 | 75.50±32.93^*^ | 94.00±34.54^*^ |
| Shoulder adduction | CRT+BWS-TCY | 14.21±7.98 | 22.00±6.40 | 27.17±5.71^*^ | 33.69±5.45^*#^ |
|  | CRT | 13.16±7.01 | 19.29±6.25 | 22.65±5.96 | 25.90±6.20 |
|  | CRT+RAT | 15.23±9.74 | 20.27±7.76 | 23.87±8.87 | 28.23±7.09^*^ |
| Shoulder external rotation | CRT+BWS-TCY | 20.97±12.18 | 33.45±9.79 | 44.03±10.94^*^ | 53.55±9.38^*^ |
|  | CRT | 18.00±11.51 | 29.13±10.97 | 36.94±11.76 | 45.45±13.84 |
|  | CRT+RAT | 22.40±13.38 | 33.77±11.88 | 40.67±11.62 | 48.53±12.31 |
| Shoulder internal rotation | CRT+BWS-TCY | 24.52±13.15 | 37.97±9.83 | 46.21±10.26^*^ | 60.34±9.81^*#^ |
|  | CRT | 21.32±12.29 | 33.29±10.09 | 38.74±11.32 | 45.91±13.69 |
|  | CRT+RAT | 26.13±14.51 | 37.53±11.79 | 43.30±12.75 | 51.57±13.30 |
| Elbow flexion | CRT+BWS-TCY | 28.97±29.49 | 59.45±26.51^*^ | 77.30±27.70^*^ | 103.93±28.69^*^ |
|  | CRT | 27.26±24.73 | 47.13±24.78 | 58.48±27.02 | 72.74±33.39 |
|  | CRT+RAT | 33.00±32.85 | 55.10±30.01 | 70.83±29.77 | 91.57±31.43^*^ |
| Forearm pronation | CRT+BWS-TCY | 28.59±14.09 | 38.52±11.33 | 48.79±11.93^*^ | 61.62±14.05^*^ |
|  | CRT | 25.68±12.96 | 34.81±9.89 | 41.90±11.48 | 50.65±13.79 |
|  | CRT+RAT | 30.03±14.35 | 38.57±11.28 | 46.33±13.09 | 54.80±15.14 |
| Forearm supination | CRT+BWS-TCY | 20.62±12.85 | 33.72±10.10 | 43.79±10.39 | 55.83±13.28^*#^ |
|  | CRT | 18.42±11.46 | 30.45±8.57 | 38.68±10.41 | 48.06±13.75 |
|  | CRT+RAT | 21.93±13.38 | 31.43±11.60 | 40.27±13.05 | 48.20±14.39 |
| ^*^ Compared with the CRT group, the difference was statistically significant (*P*< 0.05).  ^#^ Compared with the CRT+RAT group, the difference is statistically significant. (*P*< 0.05). | | | | | |

**Table 15** Repeated measurement of Mauchly sphericity hypothesis test for MBI

| Mauchly W value | Approximate chi-square value | Significance | Degrees of freedom | Greenhouse-Greisser correction |
| --- | --- | --- | --- | --- |
| 0.540 | 52.786 | <0.001 | 5 | 0.744 |

**Table 16** Repeated measures analysis of variance of MBI in three groups

| Scourses | Sum of Squares | Degrees of freedom | Mean Square | F value | Significance | Eta Squared |
| --- | --- | --- | --- | --- | --- | --- |
| Time | 28081.49 | 2.232 | 12578.59 | 533.25 | <0.001 | 0.86 |
| Group×Time | 1416.98 | 4.465 | 317.35 | 13.45 | <0.001 | 0.24 |
| Group | 1682.47 | 2 | 841.23 | 1.812 | 0.169 | 0.04 |

**Table 17** MBI scores of three groups at different time points

| Groups | Time point | Mean | Standard deviation | Confidence Interval (%) | |
| --- | --- | --- | --- | --- | --- |
|  |  |  |  | Upper | Lower |
| CRT+BWS-TCY | before intervention | 46.38 | 14.01 | 41.05 | 51.71 |
|  | 4 weeks after intervention | 55.86 | 12.61 | 51.06 | 60.66 |
|  | 8 weeks after intervention | 66.21 | 13.54 | 61.06 | 71.36 |
|  | 12 weeks after intervention | 76.55 | 12.54 | 71.78 | 81.32 |
| CRT | before intervention | 47.74 | 9.99 | 44.08 | 51.01 |
|  | 4 weeks after intervention | 52.42 | 9.56 | 48.91 | 55.93 |
|  | 8 weeks after intervention | 59.81 | 9.41 | 56.36 | 63.26 |
|  | 12 weeks after intervention | 64.52 | 10.83 | 60.54 | 68.49 |
| CRT+RAT | before intervention | 48.33 | 10.61 | 44.37 | 52.30 |
|  | 4 weeks after intervention | 55.33 | 10.98 | 51.23 | 59.43 |
|  | 8 weeks after intervention | 63.67 | 11.29 | 59.45 | 67.88 |
|  | 12 weeks after intervention | 71.67 | 10.37 | 67.80 | 75.54 |

**Table 18** Difference between three groups after MBI intervention and before intervention (Mean±SD, score)

| Groups | Difference between 4 weeks after intervention and before intervention | Difference between 8 weeks after intervention and before intervention | Difference between 12 weeks after intervention and before intervention |
| --- | --- | --- | --- |
| CRT+BWS-TCY | 8.59±7.41 | 9.48±3.86 | 30.17±6.78 |
| CRT | 6.29±7.99 | 4.68±4.82 | 16.77±6.78 |
| CRT+RAT | 7.03±5.35 | 7.00±5.81 | 23.33±7.69 |

**Table 19** Comparison of MBI between three groups after 12 weeks of intervention

| Comparison between groups | t value | *P* value (two-tailed) | Mean Difference | Standard error value |
| --- | --- | --- | --- | --- |
| CRT+BWS-TCY *VS* CRT | 6.98 | <0.001 | 13.39 | 1.92 |
| CRT+BWS-TCY *VS* CRT+RAT | 3.32 | 0.002 | 6.84 | 2.05 |
| CRT+RAT *VS* CRT | 3.54 | 0.001 | 6.56 | 1.86 |

**Table 20** Repeated measurement of Mauchly sphericity hypothesis test for SS-QOL

| Mauchly W value | Approximate chi-square value | Significance | Degrees of freedom | Greenhouse-Greisser correction |
| --- | --- | --- | --- | --- |
| 0.712 | 29.177 | <0.001 | 5 | 0.814 |

**Table 21** Repeated measures analysis of variance of SS-QOL among three groups

| Scourses | Sum of Squares | Degrees of freedom | Mean Square | F value | Significance | Eta Squared |
| --- | --- | --- | --- | --- | --- | --- |
| Time | 25933.841 | 2.443 | 10617.002 | 392.434 | <0.001 | 0.819 |
| Group×Time | 854.587 | 4.885 | 174.929 | 6.466 | <0.001 | 0.206 |
| Group | 1914.391 | 2 | 957.196 | 2.707 | 0.072 | 0.059 |

**Table 22** SS-QOL scores of three groups at different time points

| Groups | Time point | Mean | Standard deviation | Confidence Interval (%) | |
| --- | --- | --- | --- | --- | --- |
|  |  |  |  | Upper | Lower |
| CRT+BWS-TCY | before intervention | 99.79 | 13.81 | 94.54 | 105.05 |
|  | 4 weeks after intervention | 110.34 | 11.36 | 106.02 | 114.67 |
|  | 8 weeks after intervention | 114.59 | 11.82 | 110.09 | 119.08 |
|  | 12 weeks after intervention | 127.55 | 9.46 | 123.95 | 131.15 |
| CRT | before intervention | 99.58 | 10.65 | 96.56 | 106.44 |
|  | 4 weeks after intervention | 104.61 | 8.75 | 101.40 | 107.82 |
|  | 8 weeks after intervention | 108.84 | 8.27 | 105.81 | 111.87 |
|  | 12 weeks after intervention | 117.32 | 6.72 | 114.86 | 119.79 |
| CRT+RAT | before intervention | 99.37 | 13.02 | 94.51 | 104.23 |
|  | 4 weeks after intervention | 109.37 | 9.45 | 105.83 | 112.89 |
|  | 8 weeks after intervention | 111.23 | 9.89 | 109.75 | 113.89 |
|  | 12 weeks after intervention | 124.77 | 9.38 | 121.14 | 125.06 |
