# Supplementary material for: Effects of body weight-supported Tai Chi Yunshou training on upper limb motor function in stroke patients: A three-arm parallel randomized controlled trial
Source: PLoS One. 2025 Jan 9;20(1):e0314025. doi: 10.1371/journal.pone.0314025 (PMC11717223; doi:10.1371/journal.pone.0314025)
Supplement: S1 File — (DOCX) [file pone.0314025.s003.docx]

**Trial protocol**

**Project summary**

**Objectives:** To form a unique body weight support-Tai Chi Yunshou (BWS-TCY) training method, apply it to the treatment of upper limb dysfunction after stroke, and provide a new safe and effective treatment method for the clinic.

**Methods and analysis:** A single-blinded randomized controlled trial will be conducted on 93 stroke patients. The patients will be randomly allocated into three groups: 1) conventional rehabilitation therapy (CRT) group; 2) CRT+BWS-TCY group; 3) CRT+ traditional RAT group. For the CRT group, participants will receive CRT treatments for 1 hour per day. For the CRT+BWSTY group, participants will receive 30-mins CRT treatments and 30-mins BWSTY training. For the CRT+RAT group, patients will receive 30-mins CRT treatments and 30-mins RAT training. All the interventions will be performed 5 days per week for a total of 12 weeks. The upper-limb functions will be assessed before and after the interventions using a series of rating scales and objective indicators.

**Expected results:** The 12-week BWS-TCY intervention may be effective in improving upper limb motor function.

**General information**

**Protocol title:** Body Weight Support-Tai Chi Yunshou Training for Improving the Upper-extremity Function of Stroke Patients: Protocol for A Randomized Controlled Study.

**Founding:** The Shanghai Municipal Health and Health Commission Chinese Medicine Research Project (Grant No. 2020LP004), Fujian Provincial Clinical Medical Research Center for First Aid and Rehabilitation in Orthopaedic Trauma(Grant No. 2020Y2014), Medical Discipline Construction Project of Pudong New Area Commission of Health and Family Planning (Grant No. PW2022A-71), and Excellent Young Medical Talents Training Program of Pudong New District Health Commission (Grant No. PWRq2020-13).

**Researchers:** Liying Zhang^1,2^, Huanxia Zhou^1^, Yan Lu^1^, Wangsheng Liao^2^, Jiening Wang^1^, Naizhen Wang^2^, Xiaoming Yu^1^

^1^Department of Rehabilitation, The Seventh People’s Hospital Affiliated to Shanghai University of Traditional Chinese Medicine, 200137 Shanghai, China

^2^Department of Rehabilitation, Fuzhou Second Hospital, 350007 Fujian, China

**Corresponding authors**: Xiaoming Yu, Email: [278828225@qq.com](mailto:278828225@qq.com) ; Naizhen Wang ; Email: [18959115002@189.cn](mailto:18959115002@189.cn)

**Background**

Stroke is an acute cerebrovascular disease caused by the sudden rupture or occlusion of blood vessels. It is divided into two main types: ischemic stroke, which accounts for 87% of cases, and hemorrhagic stroke, which accounts for 13%^[1]^. Ischemic stroke occurs when there is ischemia and hypoxia in the brain tissue around the blood vessel supply area, leading to a disruption of local blood supply and resulting in neurological deficits^[2]^. Hemorrhagic stroke is characterized by spontaneous intracranial hemorrhage, including cerebral hemorrhage and subarachnoid hemorrhage. It is typically caused by arterial rupture due to non-traumatic factors^[3, 4]^. Stroke is the second most common cause of death and the leading cause of disability worldwide^[5, 6]^. According to statistics from the World Health Organization, approximately 15 million people worldwide suffer from stroke every year. Out of these, more than 5 million people die from stroke and another 5 million people experience permanent severe disabilities^[7]^. Moreover, stroke often leads to severe complications in patients, including neuropsychiatric disorders and impairment of motor, sensory, and cognitive abilities^[8, 9]^. Among long-term stroke survivors, 70%-80% of patients will have various types of functional impairments^[10]^. This includes 48% with hemiplegia^[11]^, 22% who are unable to walk, and 24%-53% who are partially or completely dependent on daily life activities^[12]^. Additionally, 55-75% of them still experience upper limb dysfunction 3-6 months after the stroke onset^[13]^. This dysfunction is characterized by abnormal postures such as scapula withdrawal and sinking, shoulder joint flexion, adduction, internal rotation, elbow joint flexion, forearm supination, finger flexion, etc. Furthermore, 37% of patients have varying degrees of upper limb fine motor impairment^[14]^. Unilateral upper limb dyskinesia is a frequently observed complication^[15, 16]^. Individuals experiencing upper limb dysfunction often exhibit restricted joint movement, muscle contraction difficulties, and coordination disorders^[17]^. Following a stroke, upper limb dysfunction can greatly impede daily activities like eating, dressing, and washing^[18, 19]^. This limitation increases patients' reliance on others and negatively impacts their long-term quality of life^[20]^. Consequently, rehabilitating upper limb dysfunction becomes imperative in enhancing their functional capabilities.

Different rehabilitation techniques and various therapies can be used to restore upper limb function. One such technique is repetitive transcranial magnetic stimulation, a non-invasive brain nerve modulation technology that regulates the cortical excitability of cranial nerves to promote the recovery of upper limb motor function^[21, 22]^. Another technique, task-oriented bilateral training, focuses on training both the healthy and affected sides together^[23, 24]^. This helps the affected side imitate the movement pattern of the healthy side, stimulating the memory of corresponding muscles on the affected side and promoting the recovery of motor functions. Virtual reality technology creates a simulation environment that enables virtual interaction in sight, hearing, touch^[25]^. This technology fully engages patients in training and induces neuroplasticity through repetitive training, enhancing brain movement feedback. Mirror therapy uses visual feedback to compensate for reduced or missing sensory input in the affected upper limb and establish connections between limbs^[26, 27]^. By converting visual information into active behavior, the mirror neuron system is activated, promoting movement. Robot Assistant Training (RAT) integrates multiple disciplines such as rehabilitation medicine, robotics, situational interaction technology, and control engineering. It is based on the principles of neuroplasticity and motor relearning technology, and offers the advantages of quantification, individualization, and repeatability^[28, 29]^. Therapies that involve high-intensity repetitive tasks, like RAT, have been found to be highly effective in restoring upper limb function^[30]^. These therapies offer benefits such as high-intensity repetitive training, good visual feedback, and gravitational support^[31]^. However, it is important to note that RAT compensates for the affected upper limb through gravity, which can pose challenges to the patient's compliance during the exercise and consequently impact the treatment results^[32]^. Therefore, body weight support (BWS) may not be the most suitable option for long-term rehabilitation of upper limb dysfunction after a stroke and for improving the mental health of patients. Tai Chi is a traditional Chinese aerobic exercise that involves whole-body movements, including limb wrapping, to help patients regain lost neuromuscular functions^[33]^. Specifically, Tai Chi Yunshou (TCY) is a low-impact, moderate-intensity exercise that focuses on upper limb movement training. Studies have shown that TCY is effective in improving stroke stability, endurance, coordination, and motor function^[34-36]^. What sets TCY apart from other exercise interventions is its emphasis on high coordination of the upper limbs, complex motor control, and hand-eye coordination. This type of exercise activates the cerebral cortex and brain regions to a greater extent, leading to long-term enhancement or remodeling of functional connections in the brain^[36]^. However, completing TCY exercises requires better motor functions, such as muscle strength (Lovett >2) and joint range of motion^[37]^. Additionally, TCY exercise is generally suitable for patients in the late stage of stroke (Brunnstrom stage >3)^[35]^. Unfortunately, these abilities are nearly impossible for most early-stage stroke patients. Therefore, it is crucial to develop interventions that are simple and not limited by functional impairment. These interventions should be included in current stroke rehabilitation programs to enable patients to persist in training and obtain sustained benefits from treatment.

Previous studies have demonstrated that body weight supported Tai Chi gait training, using a suspension device within a balance bar, can enhance lower limb motor function and balance in early-stage stroke patients^[38, 39]^. However, this type of training typically requires the assistance of two therapists simultaneously, which can be time-consuming and labor-intensive. Rehabilitation robots, equipped with exoskeletons and robotic arms, have the capability to provide auxiliary, resistance, and passive training. These robots can also offer gravity compensation and have been shown to offer maximum flexibility in weight support and control strategies^[40]^.

The effectiveness of TCY in the late stage of stroke for upper limb rehabilitation has been confirmed by previous studies^[35, 41]^. However, according to the guidelines of the American Stroke Association, early rehabilitation intervention leads to better outcomes^[42]^. Therefore, our research focuses on exploring the use of TCY in the early stage of stroke. Previous studies have demonstrated that weight-reduced Tai Chi footwork training, utilizing a suspension device within a balance bar, improves lower limb motor function and balance in patients with early-stage stroke^[38, 39]^. By incorporating Tai Chi with suspension devices, patients who are unable to fully bear weight can initiate training promptly. However, this training method requires the presence of at least two therapists simultaneously, resulting in time and labor consumption. Rehabilitation robots, on the other hand, offer gravity compensation and are user-friendly^[40]^. Rehabilitation robotic exoskeletons and joysticks not only provide gravity compensation but also offer power-assisted training, resistance training, and passive training. The exoskeleton provides maximum flexibility in terms of weight support and control strategies, making it easily adaptable and usable in clinical settings.

Based on the aforementioned benefits, we have developed a program utilizing a rehabilitation robot to facilitate the completion of TCY movements by driving the affected upper limb with the robot's mechanical arm. Therefore, BWS-TCY may be a suitable exercise for enhancing upper limb function in stroke patients due to its unique exercise method. Firstly, participants are required to maintain stability in their upper limbs during TCY in order to ensure smooth movements, making TCY an effective way to stimulate muscle contraction in the upper limb. Secondly, patients face challenges in controlling their speed and frequently adjusting incline angles during training. By performing TCY, coordinated movements can be trained and the flexibility of upper limb joints can be improved. Moreover, patients are required to recall and reproduce these movements during training, which can enhance their cognitive abilities, hand-eye coordination, and sense of realism. Lastly, the robot system provides vivid and engaging animations as visual feedback to enhance patient motivation during training. Therefore, TCY holds great potential in promoting the recovery of upper limb function and improving the mental well-being of stroke patients. The use of BWS-TCY can facilitate better and faster learning of TCY movements, enabling stroke survivors to independently practice at home after discharge without the need for professional guidance or supervision. Enhancing the ability of stroke survivors to carry out their own rehabilitation plans anytime and anywhere should also be considered an important rehabilitation goal.

We will propose a novel intervention using BWS-TCY to test whether it has a better rehabilitation effect than RAT in stroke patients. This study will include three groups: conventional rehabilitation treatment (CRT) group, CRT+BWS-TCY group, and CRT+ RAT group. We will hypothesize that the three groups would show differential improvements in upper limb motor function, motor control, and joint range of motion. The findings of this study will contribute to optimizing the existing rehabilitation treatment process by integrating rehabilitation intervention, aiming to intervene in rehabilitation treatment earlier and more effectively. Additionally, it will provide stroke patients with a reference for choosing rehabilitation training programs.

Study goals and objectives

**Study design**

This study will be a single-center, three-arm, parallel-group, assessors-blind randomized controlled trial. All the patients will be informed of the study content before the subject recruitment. The patients who meet the inclusion criteria and agree to participate in the study will sign the informed consent. As shown in the study flow chart (**Fig.1**), the patients will be randomly allocated into 3 groups with equal sample size: 1) CRT group; 2) CRT+RAT group; 3) CRT+ BWS-TCY group. The rehabilitation interventions will last 4 weeks. Patients’ upper-limb functions will be assessed using rating scales before and after the interventions.


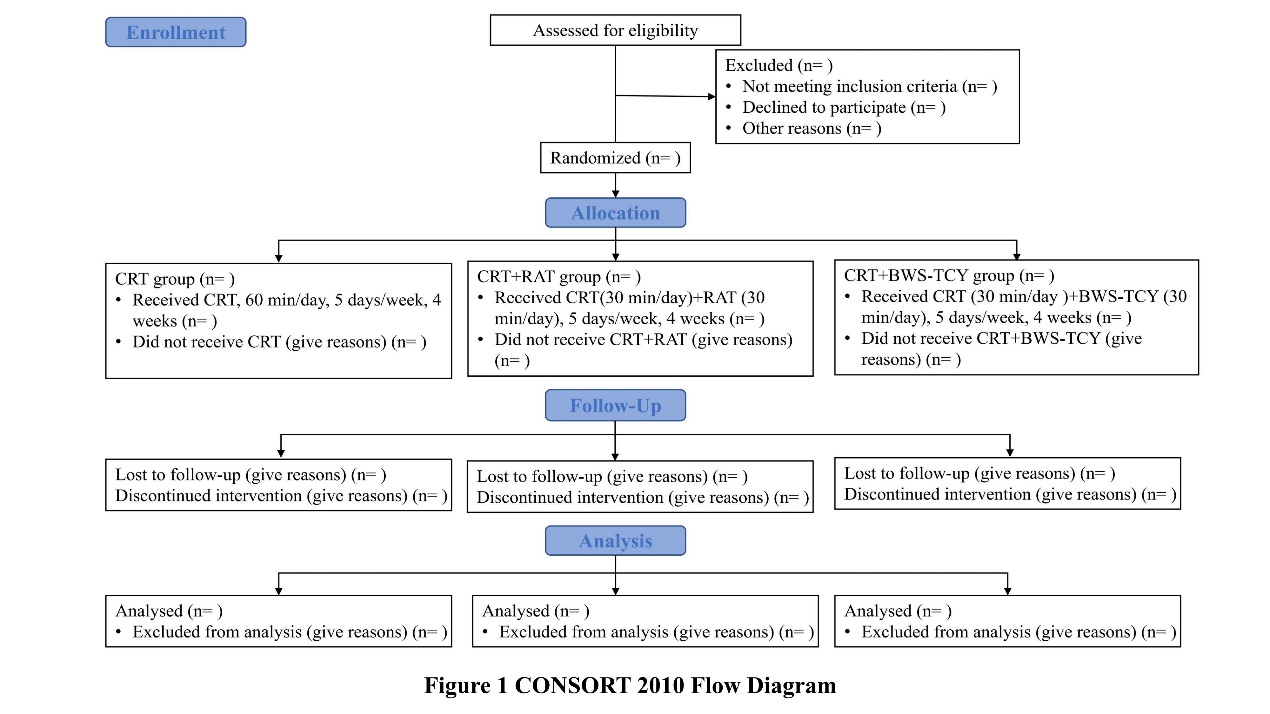


**Methodology**

**1 Study setting:** From August 2022 to Match 2023, patients will be screened and recruited by the Department of Neurological Rehabilitation and Neurology in the Seven People’s Hospital of Shanghai via reviewing their electronic medical records.

**2 Eligibility criteria**

**2.1 Inclusion criteria**

1. Presence of stroke documented by CT or MRI, including ischemic and hemorrhagic stroke;
2. Subacute stage of stroke (first onset, duration of disease within 6 months);
3. Ability to sit and balance without upper limb support, and able to tolerate at least half an hour of training or testing;
4. Brunnstrom classification of the patient's upper limb is ≤4;
5. Stable blood pressure (less than 160/100 mmHg);
6. Good cognitive ability (Mini-Mental State Examinatione score ≥22)^[43]^;
7. Good muscle tone (modified Ashworth classification < level 2);
8. No severe visual impairment or visual field defect;
9. Age between 35-80 years old, with no gender limit.

**2.2 Exclusion criteria**

1. Presence of severe bone and joint disease, muscle disease, or other neurological diseases, or a history of upper limb surgery;
2. History of skull fracture and/or severe head injury; presence of aphasia, hearing impairment, or other communication difficulties that hinder normal communication with others;
3. Manifestation of obvious shoulder pain, with a pain score at rest exceeding 5^[44]^;
4. Duration of the disease exceeding 6 months since onset;
5. Severe visual or hearing impairment that hampers the ability to cooperate with training;
6. Coexistence of severe liver, kidney, heart, lung, and blood disorders, as well as other systemic diseases;
7. Participation in other ongoing clinical studies.

**2.3 Elimination and discontinuation criteria**

1. Patients who provided incomplete information, leading to missing data on the main outcome indicators;
2. Patients who provided false information;
3. Patients with other systemic diseases that made them unsuitable to continue participating in the study;
4. Patients who experienced sudden illness or adverse events during the trial intervention period and were unable to continue or participate in the training;
5. Participants who voluntarily withdrew from the study;
6. Participants who experienced adverse events.

**3 Sample size**

We utilized G*Power software (v3.1.9.2, University Dusseldorf, Germany; available for download from http://www.psychologie.hhu.de) to determine the sample size for our study. The primary outcome evaluation index considered in this study was FMA-UE. Prior to commencing the formal experiment, 15 patients who met the inclusion criteria were divided into groups in a 1:1:1 ratio. Sample size calculation was conducted for the main objective, which focused on the treatment effect 4 weeks after surgery. The results of the preliminary experiment revealed the following mean ± standard deviation values after 4 weeks of intervention: TCY+CRT treatment (35.5±9.5), RAT+CRT treatment (34.28±9.58), and CRT alone (29.00±10.2). Based on the G*power two-factor repeated measures analysis of variance (ANOVA) F test, a total sample size of 84 cases was determined for a two-tailed test with a power of 80% and a significance level of 5% (alpha error). Considering a 1:1:1 allocation ratio and accounting for a 10% dropout rate, we estimated that a final total of 93 patients (31 in each group) would be required.

**4 Randomized grouping and allocation concealment**

In this study, a total of 93 subjects will be evenly divided into 3 groups, with 31 cases in each group. The groups will be named as follows: BWS-TCY group, CRT group, and RAT group. Subject randomization will be performed by an external professional statistician. After baseline testing, each participant received an envelope containing a randomly assigned serial number to determine their group. The random sequence will be generated by an independent professional statistician using SPSS software (IBM Corp., IBM SPSS Statistics, V25, Armonk, NY, USA), with the random number seed set to 20210608. Throughout this process, the statisticians, outcome assessors, and data analysts will be blinded to the study's recruitment, intervention, and evaluation. The randomization assignments ill be placed in sealed opaque envelopes by an independent researcher who will be also blinded to the trial. The identity of each participant is represented by a serial number, which is numbered according to their entry into the group. The letters 'A', 'B', and 'C' are used to represent the assigned groups, with 'A' corresponding to the BWS-TCY group, 'B' to the CRT group, and 'C' to the RAT group. The randomization process will be conducted by an independent researcher. Three rehabilitation therapists will be responsible for recruiting and assigning participants to conduct the intervention. Due to significant differences between the groups, neither the researchers nor the therapists will be blinded. The study will be designated as open-label, so the patients will be not blinded.

**5 Upper limb rehabilitation robot equipment and BWS-TCY program design**

The upper limb rehabilitation robot will use for BWS-TCY training and RAT training is Fourier Intelligence Co., Ltd.'s product called Fourier Arm Motus EMU. It will be a three-dimensional upper limb rehabilitation robot that operates on terminal control. The robot offers real-time, dynamic compensation for gravity and includes various games for upper limb training. These games involve activities like stretching the affected side's upper limb to musical notes in different directions (referred to as the 'grabbing the musical notes' game), simulating fishing actions and putting fish into boxes of different colors (known as the 'fishing game'), online imitation of table tennis with teammates (known as the 'table tennis game'), and activity training for various joints of the upper limbs.

BWS-TCY training utilizes the EMU robot's robotic arm to guide the affected upper limb in completing TCY movements. To facilitate this, a modified set of procedures was developed. The program consists of two modules: the first module focuses on generating customized TCY motion trajectories based on the specific characteristics of each patient, which are then saved for future training sessions. The second module is the training module, where the saved TCY trajectory can be selected and different modes (passive, assisted, and resistance) can be chosen based on the severity of upper limb impairment. The system also offers adjustable gravity compensation, as well as customizable time and range of motion. During training, the system computer simultaneously plays TCY action videos and soothing music.

**6 Interventions**

All patients will receive rehabilitation intervention in addition to routine medical treatment and daily care in the hospital. The patient's safety status will be continuously recorded throughout the intervention. The rehabilitation intervention will last for 12 weeks, with sessions of 60 minutes per day, 5 days per week.

**6.1 Intervention plan for CRT group**

In the CRT group, patients will receive 60 minutes of CRT treatment every day, will divide into two 30-minute sessions in the morning and afternoon. The training primarily focuses on the affected side and involves physical therapy, occupational therapy, and rehabilitation care. The training program includes various activities such as passive joint exercises, weight training with the assistance of the healthy hand, anti-spasm model training, functional activity series training for shoulder, elbow, and wrist joints, finger function and fine movement rehabilitation training, proper limb positioning, daily living ability training, turning over in bed training, balance training, and therapies such as proprioceptive neuromuscular stimulation technology therapy and Rood therapy.

**6.2 Intervention plan for RAT+CRT group**

After 30 minutes of CRT, the RAT training session began. The patient is seated in front of a computer and next to a robot with a mechanical arm. The height of the robotic arm is adjusted to be at the same level as the patient's shoulders. The patient is instructed to relax naturally and look straight at the computer screen in front of them. The affected forearm of the patient is secured to the handle of the robotic arm. The therapist then selects upper limb games will base on the patient's preferences and recommendations. Before entering the game interface, the therapist adjusts parameters such as gravity compensation value, training mode (active, assisted, or passive), training time, and music. In the note grabbing game, the patient is required to touch notes from different directions using the affected upper limb and count the number of touches. In the fishing game, the robot arm simulates a real fishing scene and places fish hooks on small fish of different colors that constantly change positions. The small fish are then placed in corresponding color fish frames on both sides. In the table tennis game, the patient can compete online with patients from different locations, imitate real movements, hit the ball back from the opponent, and the competition is recorded. For upper limb joint activity training, the patient can initially passively complete the movements following the system's instructions (this step can be skipped if already proficient). Once proficient, the patient can select a training mode will base on the condition of their upper limbs. The joints will train include 8 movements of the shoulder joint (forward flexion, posterior extension, abduction, adduction, external rotation, internal rotation, horizontal abduction, horizontal adduction), as well as elbow flexion and extension, and forearm pronation and supination.

**6.3 Intervention plan for BWS-TCY+CRT group**

In the CRT+BWS-TCY group, patients undergo 30 minutes of CRT treatment and 30 minutes of BWS-TCY training daily. During BWS-TCY training, patients will watch videos and learned TCY movements with the assistance of professionally trained therapists. They will be required to skillfully apply these movements. The exoskeleton rocker of the rehabilitation robot, which can customize the TCY movement trajectory, helped the patients with restricted limbs to complete TCY movements. The training process will involve the following steps: 1) Sitting upright, relaxing the body naturally, keeping the head upright, and maintaining direct eye contact with the person on the screen in front. This top-down conscious guidance facilitated overall body relaxation. 2) The affected forearm will be fixed on the handle, with the hand in an extended position. 3)The patient will follow the pre-saved TCY motion trajectory, allowing the robot's mechanical arm to assist in carrying out the TCY action. This will involve moving the shoulders, elbows, and wrists together to draw a circle clockwise from top to bottom and from inside to outside. Studies have demonstrated that as the weight support ratio increases, the degree of muscle activation gradually decreases^[38]^. Therefore, the weight support ratio was set at 40% at the beginning of the 12-week intervention program. The program followed the principle of starting from easy to difficult and repeating intensively. It was divided into 5 different weight support stages: Weeks 1-3: 40%; Weeks 4-7: 30%; Weeks 8-10: 20%; Weeks 11-12: 0%.

**7 Outcome measures**

**7.1 Fugl-Meyer motor assessment- upper extremity**

The Fugl-Meyer motor assessment- upper extremity (FMA-UE) is the main index used in this study to measure upper limb motor dysfunction after stroke. It is a cost-effective clinical examination method that is widely used in stroke patients due to its reasonable design, simplicity, and ease of use^[45]^. The FMA-UE assesses reflex activities, shoulder, elbow, and wrist joint movement, as well as coordination. It consists of 8 aspects and 33 items, with each item scored on a scale of 0 to 2 points. The total score ranges from 0 to 66 points.

**7.2 Wolf Motor Function Test**

The Wolf Motor Function Test (WMFT) is a rehabilitation scale used to assess the motor function of the upper limbs in individuals who have had a stroke^[46]^. Unlike the FMA, which primarily assesses the coordination function of stroke patients, the WMFT can evaluate both impairment and the impact of training on disability^[47]^. Additionally, it can reflect the effects of various functional task trainings on patients. The test consists of 15 items, with the first six focusing on simple joint movements and the remaining nine involving compound functional movements. Each action is timed and scored based on the quality of the movement using a six-grade scale ranging from 0 to 5 points.

**7.3 Absolute angular error**

Absolute angular error (AAE) is defined as the absolute difference between the target angle and the patient's perceived elbow flexion angle. It is used to assess the patient's proprioception and motor control^[36]^. In this study, we set the target angle to 90° of elbow flexion and an angular velocity of 2°/s, as measured by the upper limb intelligent feedback training system for evaluating upper limb proprioception. During the training session, the patient wears eye masks and earmuffs to minimize any external influence on proprioception. The patient is instructed to move the affected upper limb from the vertical position to the target position, memorize the target position, take a 10-second rest, and then repeat the same movement. The difference between the target position and the measured position is recorded as the absolute error angle. A smaller absolute error angle indicates a better position sense.

**7.4 Joint Movement Angle**

Joint Movement Angle (JMA) is a method that utilizes a goniometer to measure the maximum angle of active movement in the joints of the upper limb. The measured upper limb joint movements include shoulder flexion, shoulder extension, shoulder abduction, shoulder adduction, shoulder external rotation, shoulder internal rotation, elbow flexion, forearm pronation, and forearm supination. It is important to note that a larger angle of active movement indicates better motor function.

**7.5 Modified Barthel Index**

The improvement in basic activities of daily living can be assessed using the Modified Barthel Index (MBI), a commonly used tool for evaluating the ability of stroke patients to perform daily tasks. The MBI consists of 10 tasks, which are scored based on the time and assistance required by the patient to complete them. These tasks include eating, bathing, dressing, washing and grooming, control of defecation, control of urination, using the toilet, going up and down stairs, transferring from bed to chair, and walking 45 meters on level ground. Transferring from bed to chair and walking 45 meters on level ground have the highest scores of 15 points, while grooming and bathing have a maximum score of 5 points. The other six tasks have a maximum score of 10 points each. The lowest score for each task is 0 points, and the total score ranges from 0 to 100. A lower score indicates a greater dependence on care, while a score of 60 or higher suggests the ability to take care of oneself.

**7.6 Stroke-specific quality of life**

Stroke-specific quality of life (SS-QOL) is a patient-reported prognostic indicator used to assess the health-related quality of life in stroke patients. It can also be reported as a secondary outcome. The content of SS-QOL includes 49 items distributed across 12 domains, such as energy, family roles, language, mobility, mood, personality, self-care, social roles, thinking, upper body function, vision, and work/productivity. Each domain is scored separately, with a maximum score of 5 points for each item. The scores are then calculated to obtain the total score. A higher score indicates better functioning.

**8 Statistical method**

The clinical data will follow the per-protocol (PP) analysis and intention-to-treat (ITT) analysis. PP analysis was applied to all the randomized participants who will not stop treatment prematurely, and complete 4 weeks of treatment. ITT analysis is applied for participants who have dropped out of the trial, according to the rule of last-observation-carried-forward. The normality test of the measurement data is performed using the Shapiro-Wilk method, and the conformity distribution is expressed as the mean ± standard deviation. P<0.05 is significant for the difference. Categorical data, such as gender or those expressed as frequencies (%) will be analyzed using the chi-square test or Fisher’s exact test. Perform nonparametric statistical tests on data that do not conform to the normal distribution. In addition, statistically significant differences in intra-group and inter-group data will be analyzed by repeated measurements of ANOVA. All statistical analysis will be performed using IBM SPSS version 25.0 (SPSS Inc., Chicago, IL, USA). Missing data patterns will be analyzed by missing value analysis.

**Oversight and monitoring**

This is a single-center study conducted and coordinated at Shanghai Seventh People’s Hospital. Day-to-day support for the trial will be provided by:

- Principal Investigator: Oversees trial and medical responsibilities for patients.
- Study Coordinator: Trial registration, and coordination of study visits.
- Research therapists: Identify potential recruits, obtain informed consent, and intervene per protocol.

The research team will meet every 2 weeks. There will be no trial steering committee or stakeholder or public engagement group. A data monitoring committee was not required because of the expected low safety risk for the participants.

Throughout the study, all adverse events will be recorded in the CRF and the occurrence of adverse reactions will be fully analyzed and assessed, symptomatic treatment and active management of events. Serious adverse events occurring during the study will be reported to the ethics committee within 24 hours.

The project management team will report the research progress in the form of bi-weekly research meetings. The Trial Ethics Committee will oversee the trial procedures and recommend changes to the necessary protocol for the study. In this study, the process will be reviewed by means of on-site monitoring.

**Patient and public involvement**

The original research ideas were conceived by the authors and adjusted based on input and feedback from stroke patients and rehabilitation therapists to ensure the safety and applicability of the intervention. Before the formal experiment, 4 stroke patients will be invited for BWS-TCY training. The results of this study will be used to identify the strengths and limitations of novel interventions and advocate for improvements in their design and application.

**Follow-up**

All participants will be followed up for 12 weeks after the intervention.

Data management and statistical analysis

**Quality assurance**

Prior to enrollment, patients are required to undergo evaluations for muscle strength, muscle tone, Brunnstrom stage, and Mini-Mental State Examinatione. Additionally, a general physical examination including respiration, heart rate, blood pressure, pulse, body temperature, etc. is conducted. In light of the global epidemic of the new coronavirus, all patients must undergo a nucleic acid test, with the results being accurately documented and the therapist being informed to take necessary protective measures. Any adverse events that occur during the study period are recorded on the case report form. Adverse events refer to any unfortunate medical events, such as cardiovascular events, cerebrovascular events, and falls, that take place throughout the study.

**Expected outcomes of the study**

The 12-week BWS-TCY intervention may effectively improve upper limb motor function, and the rehabilitation effect may be better than the CRT+RAT group and CRT group.

**Dissemination of results and publication policy**

The results of the study will be published in peer-reviewed scientific journals and presented at conferences and workshops within 12 months after study completion. Individuals who meet the authorship criteria will be listed as authors of the publication, as directed by the International Committee of Medical Journal Editors. BWS-TCY exercises and corresponding equipment (programs, movement trajectories, etc.) will be optimized and promoted to the majority of physiotherapists to achieve the clinical transition.

**Duration of the project**

12 weeks intervention and 12 weeks follow-up.

**Project management**

Naizhen Wang and Xiaoming Yu conceived and designed the study; Liying Zhang and Huanxia Zhou will draft the manuscript. Xiaoming Yu and Jiening Wang will revis the manuscript for important intellectual content and acquired data. Yan Lu and Wangsheng Liao will contribute to the development of methods, including preparation for participant recruitment, training, assess outcome, data manage.

**Ethics and dissemination**

All study procedures are in accordance with the Declaration of Helsinki in its current version (for details, see www.wma.net). Patients, relatives, and their representatives were given the opportunity to discuss the study protocol and utter concerns not addressed in a draft proposed at the time. Participants will be informed of the study protocol, possible risks, and other related matters before entering the study and sign the informed consent before randomization. The present study protocol was approved by the Medical Ethics Committee of Shanghai Seventh People's Hospital (NO: 2022-7th-HIRB-022). After an initial screening of patients according to inclusion and exclusion criteria, eligible volunteers who agree to participate will sign written informed consent before the intervention. The principal investigator will be responsible for the informed consent procedure. On the consent form, the participant has the right to withdraw at any time. Participants will also be asked to allow the sharing of relevant data. The test does not involve the collection of biological samples for storage.

**Informed consent forms**

**Subject informed consent form**

**Project title:** Effects of weight-loss Tai Chi Cloud Hands on upper limb motor function in stroke patients.

**Solution version number and version date:** Version number 1.0, February 1, 2022. Informed consent form version number and version date: version number 1.0, February 1, 2021.

Dear participants:

We invite you to participate in a scientific research project approved by Shanghai Seventh People’s Hospital: the effect of weight-reducing Tai Chi Cloud Hands on the upper limb motor function of stroke patients. This study will be carried out in our hospital, and 93 subjects are expected to voluntarily participate. This study has been reviewed and approved by the Ethics Committee of Shanghai Seventh People’s Hospital.

This notice will provide you with some information to help you decide whether to participate in this clinical study. Whether you participate in this study is completely voluntary, and your decision will not affect your normal diagnosis and treatment rights and treatment in this hospital. Do not worry! If you choose to participate in this study, our research team will do our best to ensure your safety and rights during the research process!

Please read this instruction carefully, and if you have any questions, please ask the researcher responsible for explaining the informed consent form to you.

In this study, upper limb motor dysfunction is one of the most common dysfunctions in stroke patients. The mechanism of upper limb motor dysfunction after stroke is closely related to the pathological decrease in excitability of the damaged cerebral cortex and the abnormal innervation of the limbs. After a stroke, the movement and sensation of the upper limbs are directly affected, resulting in a significant reduction in the quality of life. Body weight support Tai Chi Yunshou (BWS-TCY) training is to set the TCY movement trajectory in the weight loss system, allowing patients to complete TCY training while losing weight, taking into account the dual advantages of TCY and the weight loss system. This study uses functional rating scales, brain functional connectome analysis, surface electromyography, etc. to analyze the impact of BWS-TCY training on the upper limb motor function of early stroke patients, and further explores the effect of BWS-TCY on the upper limbs after stroke. The mechanism of action of motor dysfunction and the feasibility and effectiveness of early intervention provide new ideas and new methods for clinical rehabilitation treatment of upper limb motor dysfunction after stroke.

During your participation in the clinical trial, you should follow up regularly as prescribed by your doctor. If your disease changes, you should promptly notify your doctor, who will handle the matter and monitor your health status. If you withdraw from this study for any reason, there are many other treatment options available. If any new and important information becomes available during the course of this study, your doctor will notify you promptly. You have the right to ask any questions about the study at any time, and you have the right to decide to withdraw from the study at any time. At the same time, examinations and rehabilitation evaluations during treatment are free. Whether you decide to participate or refuse to participate in this study, it will not affect the treatment of your disease.

The Ethics Committee has reviewed this study as complying with the principles of the Declaration of Helsinki and meeting ethical requirements. Your medical records will be kept intact at the hospital. Your information related to this study will be kept strictly confidential and handled with strict credibility.

If you have questions related to this study, or if you have any discomfort or injury during the study, or if you have questions about the rights of participants in this study, you can contact the project leader, Ms. Zhang Liying, through mobile phone number: 18406565061. If you have any questions or doubts about the researchers during the research process, you can contact the Medical Ethics Committee of Shanghai Seventh People’s Hospital at the telephone number: 021-58670561-6642.

**Subject consent statement:**

□ I have read the above introduction about this study, and the research doctor has explained the study content to me in detail. I have no more questions about the study to ask before signing the informed consent form. On this basis, I voluntarily participate in the clinical study described in this article, and my decision is based on a full understanding of the possible risks and benefits of participating in this study. In addition, the researcher did not use deception, inducement, coercion or other means to force me to agree to participate in the study, and I know that I can unconditionally withdraw from the study at any stage.

□ Because the subject is incapacitated or has limited capacity, this informed consent will be signed by his guardian or legal representative on his behalf.

**Signature of subject/legal representative: .**

**Date: .**

**Contact information of subject/legal representative: .**

**Reference**

[1] ZHANG T, LI X, ZHAO L, et al. Development of a Core Outcome Set in the Clinical Trials of Traditional Chinese Medicine for Stroke: A Study Protocol [J]. Frontiers in medicine, 2022, 9(753138.

[2] ZHENG Y, HU Y, HAN Z, et al. Lomitapide ameliorates middle cerebral artery occlusion-induced cerebral ischemia/reperfusion injury by promoting neuronal autophagy and inhibiting microglial migration [J]. CNS neuroscience & therapeutics, 2022, 28(12): 2183-2194.

[3] BIVARD A, KLEINIG T, CHURILOV L, et al. Permeability Measures Predict Hemorrhagic Transformation after Ischemic Stroke [J]. Annals of neurology, 2020, 88(3): 466-476.

[4] LU D, HO E, MAI H, et al. Identification of Blood Circular RNAs as Potential Biomarkers for Acute Ischemic Stroke [J]. Frontiers in neuroscience, 2020, 14(81.

[5] SUN Y, WU L, ZHONG Y, et al. Single-cell landscape of the ecosystem in early-relapse hepatocellular carcinoma [J]. Cell, 2021, 184(2): 404-421 e416.

[6] GEORGAKIS M, MALIK R, BJöRKBACKA H, et al. Circulating Monocyte Chemoattractant Protein-1 and Risk of Stroke: Meta-Analysis of Population-Based Studies Involving 17 180 Individuals [J]. Circulation research, 2019, 125(8): 773-782.

[7] SCHULIEN A, YEH C, ORANGE B, et al. Targeted disruption of Kv2.1-VAPA association provides neuroprotection against ischemic stroke in mice by declustering Kv2.1 channels [J]. Science advances, 2020, 6(27):

[8] FERRO J, CAEIRO L, FIGUEIRA M. Neuropsychiatric sequelae of stroke [J]. Nature reviews Neurology, 2016, 12(5): 269-280.

[9] GAO J, LIU J, YAO M, et al. Panax notoginseng Saponins Stimulates Neurogenesis and Neurological Restoration After Microsphere-Induced Cerebral Embolism in Rats Partially mTOR Signaling [J]. Frontiers in pharmacology, 2022, 13(889404.

[10] SHE R, YAN Z, HAO Y, et al. Comorbidity in patients with first-ever ischemic stroke: Disease patterns and their associations with cognitive and physical function [J]. Frontiers in aging neuroscience, 2022, 14(887032.

[11] HU S, CUI B, MLYNASH M, et al. Stroke epidemiology and stroke policies in China from 1980 to 2017: A systematic review and meta-analysis [J]. International journal of stroke : official journal of the International Stroke Society, 2020, 15(1): 18-28.

[12] FANG M, GO A, CHANG Y, et al. Long-term survival after ischemic stroke in patients with atrial fibrillation [J]. Neurology, 2014, 82(12): 1033-1037.

[13] DELAVARAN H, AKED J, SJUNNESSON H, et al. Spontaneous Recovery of Upper Extremity Motor Impairment After Ischemic Stroke: Implications for Stem Cell-Based Therapeutic Approaches [J]. Translational stroke research, 2017, 8(4): 351-361.

[14] DAWSON J, ENGINEER N, CRAMER S, et al. Vagus Nerve Stimulation Paired With Rehabilitation for Upper Limb Motor Impairment and Function After Chronic Ischemic Stroke: Subgroup Analysis of the Randomized, Blinded, Pivotal, VNS-REHAB Device Trial [J]. Neurorehabilitation and neural repair, 2022, 15459683221129274.

[15] LANGHORNE P, COUPAR F, POLLOCK A. Motor recovery after stroke: a systematic review [J]. The Lancet Neurology, 2009, 8(8): 741-754.

[16] HANDLEY A, MEDCALF P, HELLIER K, et al. Movement disorders after stroke [J]. Age and ageing, 2009, 38(3): 260-266.

[17] TAKEBAYASHI T, TAKAHASHI K, AMANO S, et al. Robot-Assisted Training as Self-Training for Upper-Limb Hemiplegia in Chronic Stroke: A Randomized Controlled Trial [J]. Stroke, 2022, 101161STROKEAHA121037260.

[18] AHMED I, MUSTAFAOGLU R, ROSSI S, et al. Non-invasive Brain Stimulation Techniques for the Improvement of Upper Limb Motor Function and Performance in Activities of Daily Living After Stroke: A Systematic Review and Network Meta-analysis [J]. Archives of physical medicine and rehabilitation, 2023, 104(10): 1683-1697.

[19] YUAN R, QIAO X, TANG C, et al. Effects of Uni- vs. Bilateral Upper Limb Robot-Assisted Rehabilitation on Motor Function, Activities of Daily Living, and Electromyography in Hemiplegic Stroke: A Single-Blinded Three-Arm Randomized Controlled Trial [J]. Journal of clinical medicine, 2023, 12(8):

[20] ALSUBIHEEN A, CHOI W, YU W, et al. The Effect of Task-Oriented Activities Training on Upper-Limb Function, Daily Activities, and Quality of Life in Chronic Stroke Patients: A Randomized Controlled Trial [J]. International journal of environmental research and public health, 2022, 19(21):

[21] PUNDIK S, SKELLY M, MCCABE J, et al. Does rTMS Targeting Contralesional S1 Enhance Upper Limb Somatosensory Function in Chronic Stroke? A Proof-of-Principle Study [J]. Neurorehabilitation and neural repair, 2021, 35(3): 233-246.

[22] KAKUDA W, ABO M, SASANUMA J, et al. Combination Protocol of Low-Frequency rTMS and Intensive Occupational Therapy for Post-stroke Upper Limb Hemiparesis: a 6-year Experience of More Than 1700 Japanese Patients [J]. Translational stroke research, 2016, 7(3): 172-179.

[23] LI C, WONG Y, LANGHAMMER B, et al. A study of dynamic hand orthosis combined with unilateral task-oriented training in subacute stroke: A functional near-infrared spectroscopy case series [J]. Frontiers in neurology, 2022, 13(907186.

[24] COUPAR F, POLLOCK A, VAN WIJCK F, et al. Simultaneous bilateral training for improving arm function after stroke [J]. The Cochrane database of systematic reviews, 2010, 2010(4): CD006432.

[25] KIM W, CHO S, KU J, et al. Clinical Application of Virtual Reality for Upper Limb Motor Rehabilitation in Stroke: Review of Technologies and Clinical Evidence [J]. Journal of clinical medicine, 2020, 9(10):

[26] THIEME H, MORKISCH N, MEHRHOLZ J, et al. Mirror therapy for improving motor function after stroke [J]. The Cochrane database of systematic reviews, 2018, 7(7): CD008449.

[27] TSEKLEVES E, PARASKEVOPOULOS I, WARLAND A, et al. Development and preliminary evaluation of a novel low cost VR-based upper limb stroke rehabilitation platform using Wii technology [J]. Disability and rehabilitation Assistive technology, 2016, 11(5): 413-422.

[28] XIE H, LI X, HUANG W, et al. Effects of robot-assisted task-oriented upper limb motor training on neuroplasticity in stroke patients with different degrees of motor dysfunction: A neuroimaging motor evaluation index [J]. Frontiers in neuroscience, 2022, 16(957972.

[29] HARWIN W, MURGIA A, STOKES E. Assessing the effectiveness of robot facilitated neurorehabilitation for relearning motor skills following a stroke [J]. Medical & biological engineering & computing, 2011, 49(10): 1093-1102.

[30] IWAMOTO Y, IMURA T, TANAKA R, et al. Clinical Prediction Rule for Identifying the Stroke Patients who will Obtain Clinically Important Improvement of Upper Limb Motor Function by Robot-Assisted Upper Limb [J]. Journal of stroke and cerebrovascular diseases : the official journal of National Stroke Association, 2022, 31(7): 106517.

[31] FONG J, CROCHER V, TAN Y, et al. EMU: A transparent 3D robotic manipulandum for upper-limb rehabilitation [J]. IEEE International Conference on Rehabilitation Robotics : [proceedings], 2017, 2017(771-776.

[32] FONG J, CROCHER V, HADDARA R, et al. Effect Of Arm Deweighting Using End-Effector Based Robotic Devices On Muscle Activity [J]. Annual International Conference of the IEEE Engineering in Medicine and Biology Society IEEE Engineering in Medicine and Biology Society Annual International Conference, 2018, 2018(2470-2474.

[33] DESROCHERS P, KAIRY D, PAN S, et al. Tai chi for upper limb rehabilitation in stroke patients: the patient's perspective [J]. Disability and rehabilitation, 2017, 39(13): 1313-1319.

[34] LUO X, ZHOU J, ZHANG Y, et al. Effects of Tai Chi Yunshou on upper limb function and balance in stroke survivors: A protocol for systematic review and meta analysis [J]. Medicine, 2020, 99(29): e21040.

[35] XIE G, RAO T, LIN L, et al. Effects of Tai Chi Yunshou exercise on community-based stroke patients: a cluster randomized controlled trial [J]. European review of aging and physical activity : official journal of the European Group for Research into Elderly and Physical Activity, 2018, 15(17.

[36] SUZHEN J, JINXIU C, WEINI L. The effects of Tai Chi Yunshou exercises on upper extremity function in stroke patients with hemiplegia [J]. Chinese Journal of Nursing Education, 2018, 15(03): 219-222.

[37] TAO J, RAO T, LIN L, et al. Evaluation of Tai Chi Yunshou exercises on community-based stroke patients with balance dysfunction: a study protocol of a cluster randomized controlled trial [J]. BMC complementary and alternative medicine, 2015, 15(31.

[38] HUANG S, YU X, LU Y, et al. Body weight support-Tai Chi footwork for balance of stroke survivors with fear of falling: A pilot randomized controlled trial [J]. Complementary therapies in clinical practice, 2019, 37(140-147.

[39] YU X, JIN X, LU Y, et al. Effects of Body Weight Support-Tai Chi Footwork Training on Balance Control and Walking Function in Stroke Survivors with Hemiplegia: A Pilot Randomized Controlled Trial [J]. Evidence-based complementary and alternative medicine : eCAM, 2020, 2020(9218078.

[40] FONG J, CROCHER V, TAN Y, et al. Indirect Robotic Movement Shaping through Motor Cost Influence [J]. IEEE International Conference on Rehabilitation Robotics : [proceedings], 2019, 2019(977-982.

[41] JIANG L, ZHAO L, LIU Y, et al. Effectiveness of Tai Chi Yunshou motor imagery training for hemiplegic upper extremity motor function in poststroke patients: study protocol for a randomized clinical trial [J]. Trials, 2022, 23(1): 329.

[42] Correction to: Guidelines for Adult Stroke Rehabilitation and Recovery: A Guideline for Healthcare Professionals From the American Heart Association/American Stroke Association [J]. Stroke, 2017, 48(12): e369.

[43] FOLSTEIN M, FOLSTEIN S, MCHUGH P. "Mini-mental state". A practical method for grading the cognitive state of patients for the clinician [J]. Journal of psychiatric research, 1975, 12(3): 189-198.

[44] HöGG S, HOLZGRAEFE M, WINGENDORF I, et al. Upper limb strength training in subacute stroke patients: study protocol of a randomised controlled trial [J]. Trials, 2019, 20(1): 168.

[45] LIN J, HSUEH I, SHEU C, et al. Psychometric properties of the sensory scale of the Fugl-Meyer Assessment in stroke patients [J]. Clinical rehabilitation, 2004, 18(4): 391-397.

[46] WOODBURY M, GRATTAN E, LI C. Development of a Short Form Assessment Combining the Fugl-Meyer Assessment-Upper Extremity and the Wolf Motor Function Test for Evaluating Stroke Recovery [J]. Archives of physical medicine and rehabilitation, 2023, 104(10): 1661-1668.

[47] CHEN H, WU C, LIN K, et al. Measurement properties of streamlined wolf motor function test in patients at subacute to chronic stages after stroke [J]. Neurorehabilitation and neural repair, 2014, 28(9): 839-846.
