# Supplementary material for: Effects of body weight-supported Tai Chi Yunshou training on upper limb motor function in stroke patients: A three-arm parallel randomized controlled trial
Source: PLoS One. 2025 Jan 9;20(1):e0314025. doi: 10.1371/journal.pone.0314025 (PMC11717223; doi:10.1371/journal.pone.0314025)
Supplement: S2 File — (DOCX) [file pone.0314025.s004.docx]

试验方案

**项目概要**

**目的：**形成独特的体重支撑——太极云手（BWS-TCY）训练方法，将其应用于脑卒中后上肢功能障碍的治疗，为临床提供一种安全有效的新治疗方法。

**方法与分析：**将对 93 名脑卒中患者进行单盲随机对照试验。患者将被随机分为三组：1）常规康复治疗（CRT）组； 2）CRT+BWS-TCY组； 3）CRT+传统RAT组。对于CRT组，参与者每天将接受1小时的CRT治疗。对于CRT+BWSTY组，参与者将接受30分钟的CRT治疗和30分钟的BWSTY训练。对于CRT+RAT组，患者将接受30分钟的CRT治疗和30分钟的RAT训练。所有干预措施将每周进行 5 天，总共 12 周。将使用一系列评分量表和客观指标在干预前后评估上肢功能。

**预期结果：**12周的BWS-TCY干预可能有效改善上肢运动功能。

**一般信息**

**方案标题**：减重太极云手训练对脑卒中患者上肢运动功能的影响：随机对照研究方案。

**资助项目：**上海市卫生健康委中医科研项目（批准号：2020LP004）、福建省骨科创伤急救与康复临床医学研究中心（批准号：2020Y2014）、浦东新区医科建设项目卫生计生委（批准号：PW2022A-71）和浦东新区卫生健康委优秀青年医学人才培养计划（批准号：PWRq2020-13）。

**研究者:** 张丽英^1,2^, 周欢霞^1^, 陆琰^1^, 廖旺盛^2^, 王杰宁^1^, 王乃针^2^, 于小明^1^

^1^上海中医药大学附属第七人民医院康复科, 200137 上海, 中国

^2^福州市第二总医院康复科, 350007 福建, 中国

**通讯作者**: 于小明, Email: [278828225@qq.com](mailto:278828225@qq.com) ; 王乃针 ; Email: [18959115002@189.cn](mailto:18959115002@189.cn)

**背景**

脑卒中是一种因血管突然破裂或闭塞而引起的急性脑血管疾病。 主要分为两种类型：缺血性脑卒中，占病例的87%；出血性脑卒中，占病例的13%^[1]^。当血管供应区周围的脑组织出现缺血缺氧，导致局部血液供应中断并导致神经功能缺损时，就会发生缺血性脑卒中^[2]^。出血性脑卒中以自发性颅内出血为特征，包括脑出血和蛛网膜下腔出血。它通常是由非创伤性因素导致的动脉破裂引起的^[3, 4]^。脑卒中是全世界第二大常见死亡原因和导致残疾的主要原因^[5, 6]^。据世界卫生组织统计，全球每年约有1500万人患有脑卒中。其中，超过 500 万人死于脑卒中，另有 500 万人遭受永久性严重残疾^[7]^。此外，脑卒中常常导致患者出现严重并发症，包括神经精神疾病以及运动、感觉和认知能力受损^[8, 9]^。在长期脑卒中幸存者中，70%-80%的患者会出现各种类型的功能障碍^[10]^。其中 48% 患有偏瘫^[11]^，22% 无法行走，24%-53% 部分或完全依赖日常生活活动^[12]^。此外，其中55-75%的人在脑卒中发病后3-6个月仍出现上肢功能障碍^[13]^。这种功能障碍的特点是肩胛骨后缩、下沉、肩关节屈曲、内收、内旋、肘关节屈曲、前臂旋后、手指屈曲等姿势异常。 此外，37%的患者存在不同程度的上肢精细运动障碍^[14]^。单侧上肢运动障碍是一种常见的并发症^[15, 16]^。 经历上肢功能障碍的个体通常表现出关节活动受限、肌肉收缩困难和协调障碍^[17]^。 中风后，上肢功能障碍会极大地妨碍饮食、穿衣和洗涤等日常活动^[18, 19]^。这种限制增加了患者对他人的依赖，并对他们的长期生活质量产生负面影响^[20]^。因此，恢复上肢功能障碍对于增强其功能能力至关重要。

可以采用不同的康复技术和多种疗法来恢复上肢功能，其中一项技术就是重复经颅磁刺激，这是一种非侵入性的脑神经调节技术，通过调节脑神经的皮质兴奋性来促进上肢运动功能的恢复^[21, 22]^。另一种技术是以任务为导向的双边训练，重点是同时训练健康侧和受影响侧^[23, 24]^。这有助于患侧模仿健侧的运动模式，刺激患侧相应肌肉的记忆，促进运动功能的恢复。 虚拟现实技术创建了一个模拟环境，可以实现视觉、听觉、触觉上的虚拟交互^[25]^。该技术让患者充分参与训练，通过重复训练诱发神经可塑性，增强大脑运动反馈。 镜像疗法使用视觉反馈来补偿受影响上肢减少或缺失的感觉输入，并建立肢体之间的联系^[26, 27]^。通过将视觉信息转化为主动行为，镜像神经元系统被激活，促进运动。机器人辅助训练（RAT）融合了康复医学、机器人学、情景交互技术、控制工程等多学科。它基于神经可塑性和运动再学习技术的原理，具有量化、个性化和可重复性的优点^[28, 29]^。人们发现，涉及高强度重复性任务的疗法（如 RAT）对于恢复上肢功能非常有效^[30]^。这些疗法具有高强度重复训练、良好的视觉反馈和重力支持等益处^[31]^。然而，值得注意的是，RAT 通过重力补偿受影响的上肢，这可能会给患者在运动过程中的依从性带来挑战，从而影响治疗结果^[32]^。因此，减重（BWS）可能不是中风后上肢功能障碍长期康复和改善患者心理健康的最合适选择。太极拳是一种传统的中国有氧运动，涉及全身运动，包括肢体包裹，以帮助患者恢复失去的神经肌肉功能^[33]^。具体来说，太极云手（TCY）是一种低冲击、中等强度的运动，侧重于上肢运动训练。研究表明TCY可有效改善中风稳定性、耐力、协调性和运动功能^[34-36]^。TCY 与其他运动干预措施的不同之处在于它强调上肢的高度协调性、复杂的运动控制和手眼协调性。此类运动更大程度地激活大脑皮层和大脑区域，导致大脑功能连接的长期增强或重塑^[36]^。然而，完成TCY练习需要更好的运动功能，例如肌肉力量（Lovett＞2）和关节活动范围^[37]^。另外，TCY运动一般适合脑卒中晚期（Brunnstrom分期＞3期）的患者^[35]^。不幸的是，这些能力对于大多数早期中风患者来说几乎是不可能的。 因此，制定简单且不受功能障碍限制的干预措施至关重要。这些干预措施应纳入当前的中风康复计划中，以使患者能够坚持训练并从治疗中获得持续的益处。

先前的研究表明，使用平衡杆内的悬挂装置进行体重支持的太极步态训练可以增强早期中风患者的下肢运动功能和平衡能力^[38, 39]^。然而，这种类型的培训通常需要两名治疗师同时协助，这可能既耗时又费力。康复机器人配备外骨骼和机械臂，具有提供辅助、阻力和被动训练的能力。这些机器人还可以提供重力补偿，并已被证明可以在重量支撑和控制策略方面提供最大的灵活性^[40]^。

TCY在脑卒中晚期用于上肢康复的有效性已被既往研究证实^[35, 41]^。然而，根据美国中风协会的指南，早期康复干预可以带来更好的结果^[42]^。因此，我们的研究重点是探索TCY在中风早期的使用。先前的研究表明，利用平衡杆内的悬挂装置进行减重太极步法训练可以改善早期中风患者的下肢运动功能和平衡性^[38, 39]^。通过将太极拳与悬挂装置相结合，无法完全承受体重的患者可以立即开始训练。然而，这种训练方法需要至少两名治疗师同时在场，导致时间和劳动力的消耗。另一方面，康复机器人提供重力补偿并且用户友好^[40]^。康复机器人外骨骼和操纵杆不仅提供重力补偿，还提供助力训练、阻力训练和被动训练。外骨骼在重量支撑和控制策略方面提供了最大的灵活性，使其在临床环境中易于适应和使用。

基于上述优点，我们开发了一种利用康复机器人的方案，通过机器人的机械臂驱动患侧上肢，以方便完成TCY动作。因此，BWS-TCY因其独特的运动方法，可能是一种适合脑卒中患者增强上肢功能的运动。首先，要求参与者在TCY过程中保持上肢稳定，以保证动作顺利，这使得TCY成为刺激上肢肌肉收缩的有效方法。其次，患者在训练过程中面临控制速度和频繁调整倾斜角度的挑战。通过进行TCY，可以训练协调动作，提高上肢关节的灵活性。此外，患者在训练过程中需要回忆和重现这些动作，这可以增强他们的认知能力、手眼协调能力和真实感。最后，机器人系统提供生动且引人入胜的动画作为视觉反馈，以增强患者在训练期间的积极性。因此，TCY在促进中风患者上肢功能恢复、改善心理健康方面具有巨大潜力。使用 BWS-TCY 可以促进更好更快地学习 TCY 动作，使中风幸存者出院后能够在家独立练习，无需专业指导或监督。增强中风幸存者随时随地执行自己的康复计划的能力也应被视为重要的康复目标。

我们将提出一种使用 BWS-TCY 的新型干预措施，以测试它是否比 RAT 对中风患者具有更好的康复效果。本研究将包括三组：常规康复治疗（CRT）组、CRT+BWS-TCY组、CRT+RAT组。我们假设三组在上肢运动功能、运动控制和关节活动范围方面表现出不同的改善。本研究结果将有助于通过整合康复干预来优化现有的康复治疗流程，旨在更早、更有效地干预康复治疗。 此外，还可以为中风患者选择康复训练项目提供参考。

**研究设计**

这项研究将是一项单中心、三臂、平行组、评估者盲法随机对照试验。 在受试者招募前将向所有患者告知研究内容。 符合纳入标准并同意参加研究的患者签署知情同意书。 如研究流程图（Figure 1）所示，患者将被随机分为3组，样本量相等：1）CRT组； 2）CRT+RAT组； 3）CRT+BWS-TCY组。 康复干预将持续4周。 干预前后将使用评分量表评估患者的上肢功能。


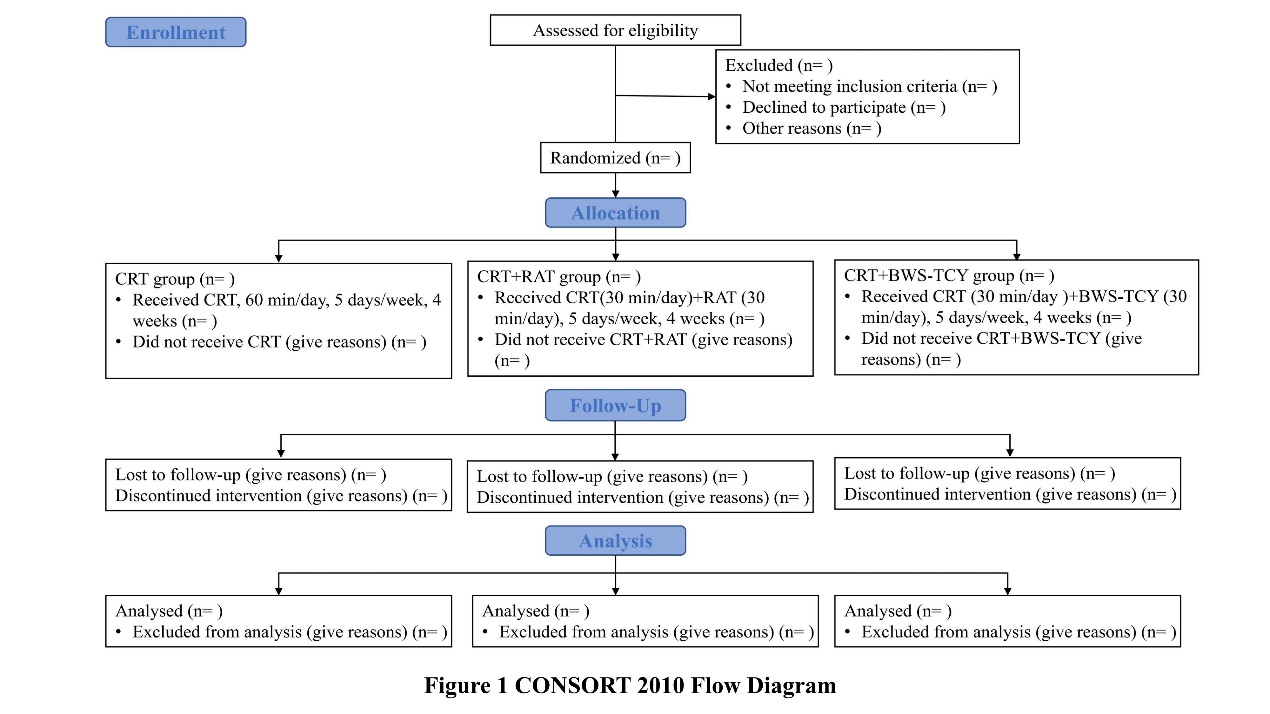


**方法**

**1 研究设置**：2022年8月至2023年3月，上海市第七人民医院神经康复科和神经内科通过查阅电子病历筛选和招募患者。

**2 资格标准**

**2.1 纳入标准**

1）经CT 或 MRI 检查的脑卒中存在，包括缺血性和出血性脑卒中；。

2）能够在没有上肢支撑的情况下坐立并保持平衡，并能够耐受至少半小时的训练或测试； 3）患者上肢Brunnstrom分级≤4级；

4) 血压稳定（低于160/100 mmHg）；

5）良好的认知能力（简易精神状态检查评分≥22）[45]；

6）良好的肌张力（改良Ashworth分类&lt;2级）；

7）无严重视力障碍或视野缺损；

8）年龄35-80岁之间，性别不限。

**2.2 排除标准**

1）有严重骨关节疾病、肌肉疾病或其他神经系统疾病，或有上肢手术史；

2) 颅骨骨折和/或严重头部损伤史；存在失语症、听力障碍或其他妨碍与他人正常沟通的沟通困难；

3）表现为明显的肩部疼痛，静息时疼痛评分超过5分^[43]^；

4）发病后病程超过6个月；

5) 视力、听力严重障碍，妨碍配合训练的；

6）同时患有严重的肝、肾、心、肺、血液疾病以及其他全身性疾病；

7) 参与其他正在进行的临床研究。

**2.3 退出和终止标准**

1）患者提供的信息不完整，导致主要结局指标数据缺失；

2）患者提供虚假信息；

3) 患有其他全身性疾病不适合继续参加研究的患者；

4）试验干预期间出现突发疾病或不良事件而无法继续或参加培训的患者；

5) 自愿退出研究的参与者；

6) 经历过不良事件的参与者。

**3 样本量计算**

我们利用 G*Power 软件（v3.1.9.2，德国杜塞尔多夫大学；可从 http://www.psychologie.hhu.de 下载）来确定我们研究的样本量。 本研究考虑的主要结局评价指标为FMA-UE。 在开始正式实验之前，将符合纳入标准的15名患者按照1:1:1的比例进行分组。 主要目标进行样本量计算，重点关注术后4周的治疗效果。初步实验结果显示干预4周后的平均值±标准差值：TCY+CRT治疗（35.5±9.5）、RAT+CRT治疗（34.28±9.58）和单独CRT（29.00±10.2）。 基于G*power双因素重复测量方差分析（ANOVA）F检验，确定总样本量84例，进行双尾检验，检验功效为80%，显着性水平为5%（α错误）。 考虑到 1:1:1 的分配比例和 10% 的退出率，我们估计最终总共需要 93 名患者（每组 31 名）。

**4 随机分组与分配隐藏**

在本研究中，共有93名受试者将被平均分为3组，每组31例。 这些组将被命名如下：BWS-TCY组、CRT组和RAT组。 受试者随机化将由外部专业统计学家进行。 基线测试后，每个参与者都会收到一个信封，其中包含随机分配的序列号，以确定他们的组别。 随机序列将由独立专业统计学家使用 SPSS 软件（IBM Corp., IBM SPSS Statistics, V25, Armonk, NY, USA）生成，随机数种子设置为 20210608。 在整个过程中，统计学家、结果评估员，数据分析师将对研究的招募、干预和评估视而不见。 随机分配将由一名独立研究人员放入密封的不透明信封中，该研究人员对试验也将不知情。 每个参与者的身份由一个序列号表示，该序列号根据他们进入组的情况进行编号。 字母“A”、“B”和“C”用于表示分配的组，其中“A”对应 BWS-TCY 组，“B”对应 CRT 组，“C”对应 RAT 组。 随机化过程将由独立研究人员进行。 三名康复治疗师将负责招募和分配参与者进行干预。 由于各组之间存在显着差异，研究人员和治疗师都不会被蒙蔽。 该研究将被指定为开放标签，因此患者不会被蒙蔽。

**5 上肢康复机器人设备及BWS-TCY程序设计**

用于BWS-TCY训练和RAT训练的上肢康复机器人是傅里叶智能有限公司的产品，名为Fourier Arm Motus EMU。 它将是一个在终端控制下运行的三维上肢康复机器人。 该机器人提供实时、动态的重力补偿，并包括各种上肢训练游戏。 这些游戏包括将患侧上肢向不同方向拉伸音符（称为“抓住音符”游戏）、模拟钓鱼动作并将鱼放入不同颜色的盒子中（称为“钓鱼游戏”）等活动。 ）、与队友在线模仿乒乓球（俗称“乒乓球比赛”）、上肢各关节的活动训练。

BWS-TCY训练利用动车组机器人的机械臂引导受影响的上肢完成TCY动作。 为了促进这一点，开发了一套修改后的程序。 该程序由两个模块组成：第一个模块侧重于根据每个患者的具体特征生成定制的 TCY 运动轨迹，然后将其保存以供将来的培训课程使用。 第二个模块是训练模块，可以选择保存的TCY轨迹，并可以根据上肢损伤的严重程度选择不同的模式（被动、辅助、阻力）。 该系统还提供可调节的重力补偿，以及可定制的时间和运动范围。 训练时，系统电脑同时播放TCY动作视频和舒缓的音乐。

**6 干预方法**

所有患者除了在医院进行常规医疗和日常护理外，还将接受康复干预。 在整个干预过程中，患者的安全状态将被持续记录。 康复干预将持续 12 周，每天 60 分钟，每周 5 天。

**6.1 CRT组干预计划**

在CRT组中，患者每天将接受60分钟的CRT治疗，分为上午和下午两个疗程，各30分钟。培训主要针对患侧，包括物理治疗、职业治疗和康复护理。训练内容包括被动关节练习、健手辅助负重训练、抗痉挛模型训练、肩、肘、腕关节功能活动系列训练、手指功能及精细动作康复训练、适当运动训练等。 肢体定位、日常生活能力训练、床上翻身训练、平衡训练以及本体感觉神经肌肉刺激技术疗法、Rood疗法等疗法。

**6.2 RAT+CRT组干预计划**

CRT 30分钟后，RAT训练开始。患者坐在电脑前，旁边是一个带有机械臂的机器人。 将机械臂的高度调整至与患者肩膀处于同一水平。指导患者自然放松并直视面前的电脑屏幕。 患者受影响的前臂固定在机械臂的手柄上。然后治疗师将根据患者的喜好和建议选择上肢游戏。在进入游戏界面之前，治疗师调整重力补偿值、训练模式（主动、辅助或被动）、训练时间、音乐等参数。在抓笔记游戏中，患者需要用受影响的上肢从不同方向触摸笔记并计算触摸的次数。在钓鱼游戏中，机械臂模拟真实的钓鱼场景，将鱼钩放在不断变换位置的不同颜色的小鱼上。然后将小鱼放入两侧相应颜色的鱼框内。 在乒乓球比赛中，患者可以与来自不同地点的患者进行在线比赛，模仿真实动作，将球从对手手中击回，并记录比赛情况。 对于上肢关节活动训练，患者最初可以按照系统指令被动完成动作（如果已经熟练可以跳过此步骤）。 熟练后，患者可以根据自己上肢的情况选择训练模式。 训练的关节包括肩关节的8个动作（前屈、后伸、外展、内收、外旋、内旋、水平外展、水平内收），以及肘关节屈伸、前臂旋前和旋后。

**6.3 BWS-TCY+CRT组干预方案**

CRT+BWS-TCY组患者每天接受30分钟的CRT治疗和30分钟的BWS-TCY训练。在BWS-TCY培训期间，患者将在经过专业培训的治疗师的帮助下观看视频并学习TCY动作。 他们将需要熟练地应用这些动作。康复机器人的外骨骼摇杆可以定制TCY运动轨迹，帮助肢体受限的患者完成TCY运动。训练过程将包括以下步骤： 1）坐直，身体自然放松，保持头部直立，与前面屏幕上的人保持直接的目光接触。 这种自上而下的意识引导促进了全身放松。 2) 将受影响的前臂固定在手柄上，手处于伸展位置。 3）患者将遵循预先保存的TCY运动轨迹，让机器人的机械臂协助进行TCY动作。这将涉及到一起移动肩膀、肘部和手腕，从上到下、从内到外顺时针画一个圆圈。研究表明，随着重量支撑比的增加，肌肉激活程度逐渐降低[38]。因此，在为期12周的干预计划开始时，体重支持比例设定为40%。节目遵循由易到难、密集重复的原则。分为5个不同的重量支持阶段：第1-3周：40%；第 4-7 周：30%；第 8-10 周：20%；第 11-12 周：0%。

**7 结果测量**

**7.1 Fugl-Meyer 上肢运动功能评估**

Fugl-Meyer上肢运动功能评估（FMA-UE）是本研究用于衡量脑卒中后上肢运动功能障碍的主要指标。由于其设计合理、简单易用，是一种经济有效的临床检查方法，广泛应用于脑卒中患者^[44]^。 FMA-UE 评估反射活动、肩部、肘部和腕部关节运动以及协调性。 它由8个方面、33个条目组成，每个条目的评分范围为0至2分。 总分范围为0至66分。

**7.2 Wolf 运动功能测试**

Wolf 运动功能测试 (WMFT) 是一种康复量表，用于评估中风患者上肢的运动功能^[45]^。 与主要评估中风患者协调功能的 FMA 不同，WMFT 可以评估损伤以及训练对残疾的影响^[46]^。此外，它还可以反映各种功能任务训练对患者的效果。该测试由15个项目组成，其中前6个项目侧重于简单的关节运动，其余9个项目涉及复合功能运动。每个动作都会根据动作的质量进行计时和评分，采用 0 到 5 分的六级量表。

**7.3 绝对角度误差**

绝对角度误差 (AAE) 定义为目标角度与患者感知的肘部弯曲角度之间的绝对差。它用于评估患者的本体感觉和运动控制^[36]^。在本研究中，我们将目标角度设置为肘部屈曲90°，角速度为2°/s，通过上肢智能反馈训练系统测量来评估上肢本体感觉。在训练过程中，患者佩戴眼罩和耳罩，以尽量减少外部对本体感觉的影响。指导患者将受影响的上肢从垂直位置移动到目标位置，记住目标位置，休息10秒，然后重复相同的动作。目标位置与测量位置之间的差值记录为绝对误差角度。 绝对误差角越小，位置感越好。

**7.4 关节运动角度**

关节运动角度（JMA）是一种利用测角仪测量上肢关节主动运动最大角度的方法。 测量的上肢关节运动包括肩屈、肩伸、肩外展、肩内收、肩外旋、肩内旋、肘屈曲、前臂旋前、前臂旋后。 值得注意的是，主动运动的角度越大表明运动功能越好。

**7.5 改良Bathel指数**

日常生活基本活动的改善可以使用改良Bathel指数（MBI）来评估，该指数是评估中风患者执行日常任务能力的常用工具。 MBI 包含 10 项任务，根据患者完成任务所需的时间和协助进行评分。这些任务包括吃饭、洗澡、穿衣、洗涤和梳洗、控制排便、控制排尿、使用厕所、上下楼梯、从床上转移到椅子上以及在平地上行走 45 米。从床上转移到椅子上以及在平地上行走45米的得分最高为15分，而梳洗和洗澡的得分最高为5分。其他六项任务每项最高得分为 10 分。每项任务最低分为0分，总分为0到100分。分数较低表示对护理的依赖程度较高，而60分或以上表示有照顾自己的能力。

**7.6 卒中专用生活质量量表**

卒中专用生活质量量表（SS-QOL）是患者报告的预后指标，用于评估中风患者与健康相关的生活质量。它也可以作为次要结果报告。 SS-QOL的内容包括分布在12个领域的49个项目，例如能量、家庭角色、语言、行动能力、情绪、个性、自我保健、社会角色、思维、上半身功能、视力和工作/生产力。每个领域单独评分，每项最高分为5分。然后计算分数以获得总分。 分数越高表明功能越好。

**8 统计学方法**

临床数据遵循符合方案（PP）分析和意向治疗（ITT）分析。 PP 分析适用于所有不会提前停止治疗并完成 4 周治疗的随机参与者。根据最后观察结转的规则，ITT 分析适用于退出试验的参与者。采用Shapiro-Wilk法对计量数据进行正态性检验，合格分布表示为平均值±标准差。 P＜0.05表示 统计学差异显着。分类数据，例如性别或以频率 (%) 表示的数据，将使用卡方检验或费舍尔精确检验进行分析。 对不符合正态分布的数据进行非参数统计检验。此外，将通过重复测量方差分析来分析组内和组间数据的统计显着差异。所有统计分析都将使用 IBM SPSS 25.0 (SPSS Inc., Chicago, IL, USA)进行。将通过缺失值分析来分析缺失数据模式。

**监督和监测**

这是一项在上海市第七人民医院进行和协调的单中心研究。试验的日常支持将由以下人员提供：

• 首席研究员：监督试验和患者的医疗责任。

• 研究协调员：试验注册和研究访问的协调。

• 研究治疗师：识别潜在的招募者，获得知情同意，并按照方案进行干预。

研究团队每两周举行一次会议。不会有试验指导委员会、利益相关者或公众参与小组。由于预期参与者的安全风险较低，因此不需要数据监控委员会。

在整个研究过程中，所有不良事件都会记录在CRF中，并对不良反应的发生进行充分分析和评估，对症治疗并积极管理事件。 研究过程中发生的严重不良事件将在24小时内报告给伦理委员会。

项目管理团队将以每两周一次的研究会议的形式汇报研究进展。试验伦理委员会将监督试验程序并建议更改必要的研究方案。在本研究中，将通过现场监测的方式来审查该过程。

**患者和公众的参与**

最初的研究思路由作者构思，并根据中风患者和康复治疗师的意见和反馈进行调整，以确保干预措施的安全性和适用性。在正式实验之前，将邀请4名中风患者进行BWS-TCY培训。这项研究的结果将用于确定新颖干预措施的优点和局限性，并倡导改进其设计和应用。

**随访**

所有参与者将在干预后接受 12 周的随访。

**质量保证**

在入组之前，患者需要接受肌力、肌张力、Brunnstrom 阶段和简易精神状态检查的评估。此外，还会进行一般身体检查，包括呼吸、心率、血压、脉搏、体温等。鉴于新型冠状病毒在全球流行，所有患者都必须接受核酸检测，准确记录结果，并通知治疗师采取必要的防护措施。研究期间发生的任何不良事件均记录在病例报告表上。不良事件是指在整个研究过程中发生的任何不幸的医疗事件，例如心血管事件、脑血管事件、跌倒等。

**研究的预期结果**

12周的BWS-TCY干预可有效改善上肢运动功能，康复效果可能优于CRT+RAT组和CRT组。

**结果传播和出版政策**

研究结果将在研究完成后 12 个月内发表在同行评审的科学期刊上，并在会议和研讨会上发表。根据国际医学期刊编辑委员会的指示，符合作者身份标准的个人将被列为出版物的作者。BWS-TCY练习和相应的设备（程序、运动轨迹等）将被优化并推广给广大物理治疗师，以实现临床过渡。

**项目的持续时间**

12 周干预和 12 周随访。

**项目管理**

王乃针和于小明构思并设计了该研究；张丽英和周焕霞将起草手稿。于小明和王杰宁将修改手稿中的重要知识内容和获取的数据。陆琰和廖旺盛将致力于方法的开发，包括参与者招募、培训、评估结果、数据管理的准备。

**伦理与传播**

所有研究程序均符合当前版本的赫尔辛基宣言（有关详细信息，请参见 www.wma.net）。 患者、亲属及其代表有机会讨论研究方案并提出当时提出的草案中未解决的问题。参与者在进入研究前将被告知研究方案、可能的风险和其他相关事项，并在随机分组前签署知情同意书。本研究方案经上海市第七人民医院医学伦理委员会批准（批准号：2022-7th-HIRB-022）。根据纳入和排除标准对患者进行初步筛选后，同意参与的符合条件的志愿者将在干预前签署书面知情同意书。主要研究者将负责知情同意程序。在知情同意书上，参与者有权随时退出。参与者还将被要求允许共享相关数据。 该测试不涉及生物样本的收集和储存。

**知情同意书**

**受试者知情同意书**

项目名称：项目名称：减重太极云手对脑卒中患者上肢运动功能的影响。

方案版本号及版本日期：版本号1.0**,** 2022年2月1日

知情同意书版本号及版本日期：版本号1.0, 2021年2月1日

尊敬的患者：

尊敬的参与者：我们邀请您参与上海市批准的一项科研项目第七人民医院：减重太极云手对脑卒中患者上肢运动功能的影响。本研究将在我院进行，预计有93名受试者自愿参加。本研究已经上海市第七人民医院伦理委员会审查通过。

本通知将为您提供一些信息，帮助您决定是否参加本临床研究。您是否参加本研究完全是自愿的，您的决定不会影响您在本院正常的诊疗权利和治疗。不用担心！如果您选择参与本次研究，我们的研究团队将尽最大努力保障您在研究过程中的安全和权益！

请仔细阅读本说明，如有任何疑问，请询问负责向您解释知情同意书的研究者。

在这项研究中，上肢运动功能障碍是中风患者最常见的功能障碍之一。 脑卒中后上肢运动功能障碍的机制与受损的大脑皮层兴奋性病理性降低、肢体神经支配异常密切相关。中风后，上肢的运动和感觉受到直接影响，导致生活质量显着下降。减重太极云手（BWS-TCY）训练是在减肥系统中设定TCY运动轨迹，让患者在减重的同时完成TCY训练，兼顾双重优势。

您在参加临床试验的过程中应该按照医生的规定定期随访,疾病如有变化，应及时通知您的医生，他/她将对此作出处理并监护您的健康状况。无论何种原因，您退出本研究，有很多其它治疗的方案可代替。如在本研究进行期间，获得某些全新重要的信息，您的医生将会及时通知您。您有权在任何时间询问有关本研究的任何问题，并且您有权决定在任何时间退出本研究。同时治疗过程中的检查及康复评估均是免费的。无论您决定参加或拒绝参加本研究，都不会影响您本病的治疗。

伦理委员会已经审议此项研究是遵从赫尔辛基宣言原则的，是符合伦理要求的。您的医疗记录将完整的保存在医院。您与本研究的相关资料都将会得到严格的保密及进行严格可信的处理。

如果您有与本研究有关的问题，或您在研究过程中发生了任何不适与损伤，或有关于本项研究参加者权益方面的问题您可以通过手机号：18406565061与项目负责人张丽英老师联系。如果您有任何疑问或在研究过程中对研究人员有疑义，可以联系上海市第七人民医院医学伦理委员会，联系电话： 021-58670561-6642。

**受试者签字**

**受试者同意声明：**

□ 我已经阅读了上述有关本研究的介绍，且研究医生已向我详细地讲解了研究内容，在签署知情同意书前我已没有更多有关研究的疑惑需咨询。在此基础上，我自愿参加本文所介绍的临床研究，并且我的决定是基于对参加本研究可能产生的风险和受益充分了解。此外，研究者没有对我使用欺骗、利诱、胁迫等手段强行让我同意参加研究，并且我知道我可以在任何阶段无条件退出研究。

□该名受试者因无行为能力、限制行为能力，本知情同意由其监护人或者法定代理人代为签署。

受试者签名： 法定代理人签名：

日 期： 日 期：

受试者联系方式： 法定代理人联系方式：

**研究者声明：**

我确认已向患者解释了本研究的详细情况，特别是参加本研究可能产生的风险和收益。

研究者签名：

日 期：

研究者联系方式：

**参考文献**

[1] ZHANG T, LI X, ZHAO L, et al. Development of a Core Outcome Set in the Clinical Trials of Traditional Chinese Medicine for Stroke: A Study Protocol [J]. Frontiers in medicine, 2022, 9(753138.

[2] ZHENG Y, HU Y, HAN Z, et al. Lomitapide ameliorates middle cerebral artery occlusion-induced cerebral ischemia/reperfusion injury by promoting neuronal autophagy and inhibiting microglial migration [J]. CNS neuroscience & therapeutics, 2022, 28(12): 2183-2194.

[3] BIVARD A, KLEINIG T, CHURILOV L, et al. Permeability Measures Predict Hemorrhagic Transformation after Ischemic Stroke [J]. Annals of neurology, 2020, 88(3): 466-476.

[4] LU D, HO E, MAI H, et al. Identification of Blood Circular RNAs as Potential Biomarkers for Acute Ischemic Stroke [J]. Frontiers in neuroscience, 2020, 14(81.

[5] SUN Y, WU L, ZHONG Y, et al. Single-cell landscape of the ecosystem in early-relapse hepatocellular carcinoma [J]. Cell, 2021, 184(2): 404-421 e416.

[6] GEORGAKIS M, MALIK R, BJöRKBACKA H, et al. Circulating Monocyte Chemoattractant Protein-1 and Risk of Stroke: Meta-Analysis of Population-Based Studies Involving 17 180 Individuals [J]. Circulation research, 2019, 125(8): 773-782.

[7] SCHULIEN A, YEH C, ORANGE B, et al. Targeted disruption of Kv2.1-VAPA association provides neuroprotection against ischemic stroke in mice by declustering Kv2.1 channels [J]. Science advances, 2020, 6(27):

[8] FERRO J, CAEIRO L, FIGUEIRA M. Neuropsychiatric sequelae of stroke [J]. Nature reviews Neurology, 2016, 12(5): 269-280.

[9] GAO J, LIU J, YAO M, et al. Panax notoginseng Saponins Stimulates Neurogenesis and Neurological Restoration After Microsphere-Induced Cerebral Embolism in Rats Partially mTOR Signaling [J]. Frontiers in pharmacology, 2022, 13(889404.

[10] SHE R, YAN Z, HAO Y, et al. Comorbidity in patients with first-ever ischemic stroke: Disease patterns and their associations with cognitive and physical function [J]. Frontiers in aging neuroscience, 2022, 14(887032.

[11] HU S, CUI B, MLYNASH M, et al. Stroke epidemiology and stroke policies in China from 1980 to 2017: A systematic review and meta-analysis [J]. International journal of stroke : official journal of the International Stroke Society, 2020, 15(1): 18-28.

[12] FANG M, GO A, CHANG Y, et al. Long-term survival after ischemic stroke in patients with atrial fibrillation [J]. Neurology, 2014, 82(12): 1033-1037.

[13] DELAVARAN H, AKED J, SJUNNESSON H, et al. Spontaneous Recovery of Upper Extremity Motor Impairment After Ischemic Stroke: Implications for Stem Cell-Based Therapeutic Approaches [J]. Translational stroke research, 2017, 8(4): 351-361.

[14] DAWSON J, ENGINEER N, CRAMER S, et al. Vagus Nerve Stimulation Paired With Rehabilitation for Upper Limb Motor Impairment and Function After Chronic Ischemic Stroke: Subgroup Analysis of the Randomized, Blinded, Pivotal, VNS-REHAB Device Trial [J]. Neurorehabilitation and neural repair, 2022, 15459683221129274.

[15] LANGHORNE P, COUPAR F, POLLOCK A. Motor recovery after stroke: a systematic review [J]. The Lancet Neurology, 2009, 8(8): 741-754.

[16] HANDLEY A, MEDCALF P, HELLIER K, et al. Movement disorders after stroke [J]. Age and ageing, 2009, 38(3): 260-266.

[17] TAKEBAYASHI T, TAKAHASHI K, AMANO S, et al. Robot-Assisted Training as Self-Training for Upper-Limb Hemiplegia in Chronic Stroke: A Randomized Controlled Trial [J]. Stroke, 2022, 101161STROKEAHA121037260.

[18] AHMED I, MUSTAFAOGLU R, ROSSI S, et al. Non-invasive Brain Stimulation Techniques for the Improvement of Upper Limb Motor Function and Performance in Activities of Daily Living After Stroke: A Systematic Review and Network Meta-analysis [J]. Archives of physical medicine and rehabilitation, 2023, 104(10): 1683-1697.

[19] YUAN R, QIAO X, TANG C, et al. Effects of Uni- vs. Bilateral Upper Limb Robot-Assisted Rehabilitation on Motor Function, Activities of Daily Living, and Electromyography in Hemiplegic Stroke: A Single-Blinded Three-Arm Randomized Controlled Trial [J]. Journal of clinical medicine, 2023, 12(8):

[20] ALSUBIHEEN A, CHOI W, YU W, et al. The Effect of Task-Oriented Activities Training on Upper-Limb Function, Daily Activities, and Quality of Life in Chronic Stroke Patients: A Randomized Controlled Trial [J]. International journal of environmental research and public health, 2022, 19(21):

[21] PUNDIK S, SKELLY M, MCCABE J, et al. Does rTMS Targeting Contralesional S1 Enhance Upper Limb Somatosensory Function in Chronic Stroke? A Proof-of-Principle Study [J]. Neurorehabilitation and neural repair, 2021, 35(3): 233-246.

[22] KAKUDA W, ABO M, SASANUMA J, et al. Combination Protocol of Low-Frequency rTMS and Intensive Occupational Therapy for Post-stroke Upper Limb Hemiparesis: a 6-year Experience of More Than 1700 Japanese Patients [J]. Translational stroke research, 2016, 7(3): 172-179.

[23] LI C, WONG Y, LANGHAMMER B, et al. A study of dynamic hand orthosis combined with unilateral task-oriented training in subacute stroke: A functional near-infrared spectroscopy case series [J]. Frontiers in neurology, 2022, 13(907186.

[24] COUPAR F, POLLOCK A, VAN WIJCK F, et al. Simultaneous bilateral training for improving arm function after stroke [J]. The Cochrane database of systematic reviews, 2010, 2010(4): CD006432.

[25] KIM W, CHO S, KU J, et al. Clinical Application of Virtual Reality for Upper Limb Motor Rehabilitation in Stroke: Review of Technologies and Clinical Evidence [J]. Journal of clinical medicine, 2020, 9(10):

[26] THIEME H, MORKISCH N, MEHRHOLZ J, et al. Mirror therapy for improving motor function after stroke [J]. The Cochrane database of systematic reviews, 2018, 7(7): CD008449.

[27] TSEKLEVES E, PARASKEVOPOULOS I, WARLAND A, et al. Development and preliminary evaluation of a novel low cost VR-based upper limb stroke rehabilitation platform using Wii technology [J]. Disability and rehabilitation Assistive technology, 2016, 11(5): 413-422.

[28] XIE H, LI X, HUANG W, et al. Effects of robot-assisted task-oriented upper limb motor training on neuroplasticity in stroke patients with different degrees of motor dysfunction: A neuroimaging motor evaluation index [J]. Frontiers in neuroscience, 2022, 16(957972.

[29] HARWIN W, MURGIA A, STOKES E. Assessing the effectiveness of robot facilitated neurorehabilitation for relearning motor skills following a stroke [J]. Medical & biological engineering & computing, 2011, 49(10): 1093-1102.

[30] IWAMOTO Y, IMURA T, TANAKA R, et al. Clinical Prediction Rule for Identifying the Stroke Patients who will Obtain Clinically Important Improvement of Upper Limb Motor Function by Robot-Assisted Upper Limb [J]. Journal of stroke and cerebrovascular diseases : the official journal of National Stroke Association, 2022, 31(7): 106517.

[31] FONG J, CROCHER V, TAN Y, et al. EMU: A transparent 3D robotic manipulandum for upper-limb rehabilitation [J]. IEEE International Conference on Rehabilitation Robotics : [proceedings], 2017, 2017(771-776.

[32] FONG J, CROCHER V, HADDARA R, et al. Effect Of Arm Deweighting Using End-Effector Based Robotic Devices On Muscle Activity [J]. Annual International Conference of the IEEE Engineering in Medicine and Biology Society IEEE Engineering in Medicine and Biology Society Annual International Conference, 2018, 2018(2470-2474.

[33] DESROCHERS P, KAIRY D, PAN S, et al. Tai chi for upper limb rehabilitation in stroke patients: the patient's perspective [J]. Disability and rehabilitation, 2017, 39(13): 1313-1319.

[34] LUO X, ZHOU J, ZHANG Y, et al. Effects of Tai Chi Yunshou on upper limb function and balance in stroke survivors: A protocol for systematic review and meta analysis [J]. Medicine, 2020, 99(29): e21040.

[35] XIE G, RAO T, LIN L, et al. Effects of Tai Chi Yunshou exercise on community-based stroke patients: a cluster randomized controlled trial [J]. European review of aging and physical activity : official journal of the European Group for Research into Elderly and Physical Activity, 2018, 15(17.

[36] SUZHEN J, JINXIU C, WEINI L. The effects of Tai Chi Yunshou exercises on upper extremity function in stroke patients with hemiplegia [J]. Chinese Journal of Nursing Education, 2018, 15(03): 219-222.

[37] TAO J, RAO T, LIN L, et al. Evaluation of Tai Chi Yunshou exercises on community-based stroke patients with balance dysfunction: a study protocol of a cluster randomized controlled trial [J]. BMC complementary and alternative medicine, 2015, 15(31.

[38] HUANG S, YU X, LU Y, et al. Body weight support-Tai Chi footwork for balance of stroke survivors with fear of falling: A pilot randomized controlled trial [J]. Complementary therapies in clinical practice, 2019, 37(140-147.

[39] YU X, JIN X, LU Y, et al. Effects of Body Weight Support-Tai Chi Footwork Training on Balance Control and Walking Function in Stroke Survivors with Hemiplegia: A Pilot Randomized Controlled Trial [J]. Evidence-based complementary and alternative medicine : eCAM, 2020, 2020(9218078.

[40] FONG J, CROCHER V, TAN Y, et al. Indirect Robotic Movement Shaping through Motor Cost Influence [J]. IEEE International Conference on Rehabilitation Robotics : [proceedings], 2019, 2019(977-982.

[41] JIANG L, ZHAO L, LIU Y, et al. Effectiveness of Tai Chi Yunshou motor imagery training for hemiplegic upper extremity motor function in poststroke patients: study protocol for a randomized clinical trial [J]. Trials, 2022, 23(1): 329.

[42] Correction to: Guidelines for Adult Stroke Rehabilitation and Recovery: A Guideline for Healthcare Professionals From the American Heart Association/American Stroke Association [J]. Stroke, 2017, 48(12): e369.

[43] HöGG S, HOLZGRAEFE M, WINGENDORF I, et al. Upper limb strength training in subacute stroke patients: study protocol of a randomised controlled trial [J]. Trials, 2019, 20(1): 168.

[44] LIN J, HSUEH I, SHEU C, et al. Psychometric properties of the sensory scale of the Fugl-Meyer Assessment in stroke patients [J]. Clinical rehabilitation, 2004, 18(4): 391-397.

[45] WOODBURY M, GRATTAN E, LI C. Development of a Short Form Assessment Combining the Fugl-Meyer Assessment-Upper Extremity and the Wolf Motor Function Test for Evaluating Stroke Recovery [J]. Archives of physical medicine and rehabilitation, 2023, 104(10): 1661-1668.

[46] CHEN H, WU C, LIN K, et al. Measurement properties of streamlined wolf motor function test in patients at subacute to chronic stages after stroke [J]. Neurorehabilitation and neural repair, 2014, 28(9): 839-846.
